# Chemical kinetics in an atmospheric pressure helium plasma containing humidity†
†Data underpinning the figures in this manuscript can be found at DOI: 10.15124/1a186859-ad12-4e33-bf17-ccfcbad48ba0.


**DOI:** 10.1039/c8cp02473a

**Published:** 2018-09-13

**Authors:** Sandra Schröter, Apiwat Wijaikhum, Andrew R. Gibson, Andrew West, Helen L. Davies, Nicolas Minesi, James Dedrick, Erik Wagenaars, Nelson de Oliveira, Laurent Nahon, Mark J. Kushner, Jean-Paul Booth, Kari Niemi, Timo Gans, Deborah O'Connell

**Affiliations:** a York Plasma Institute , Department of Physics , University of York , Heslington , York YO10 5DD , UK . Email: sandra.schroeter@york.ac.uk; b LPP , CNRS , Ecole Polytechnique , UPMC Univ. Paris-Sud , Observatoire de Paris , Université Paris-Saclay , Sorbonne Universités , PSL Research University , 91128 Palaiseau , France; c Centre of Immunology and Infection , Department of Biology and Hull York Medical School , University of York , Heslington , York YO10 5DD , UK; d Synchrotron Soleil , l'Orme des Merisiers , St. Aubin BP 48 , 91192 Gif sur Yvette Cedex , France; e Department of Electrical Engineering and Computer Science , University of Michigan , 1301 Beal Ave. , Ann Arbor , Michigan 48109-2122 , USA

## Abstract

Atmospheric pressure plasmas are sources of biologically active oxygen and nitrogen species, which makes them potentially suitable for the use as biomedical devices. Here, experiments and simulations are combined to investigate the formation of the key reactive oxygen species, atomic oxygen (O) and hydroxyl radicals (OH), in a radio-frequency driven atmospheric pressure plasma jet operated in humidified helium. Vacuum ultra-violet high-resolution Fourier-transform absorption spectroscopy and ultra-violet broad-band absorption spectroscopy are used to measure absolute densities of O and OH. These densities increase with increasing H_2_O content in the feed gas, and approach saturation values at higher admixtures on the order of 3 × 10^14^ cm^–3^ for OH and 3 × 10^13^ cm^–3^ for O. Experimental results are used to benchmark densities obtained from zero-dimensional plasma chemical kinetics simulations, which reveal the dominant formation pathways. At low humidity content, O is formed from OH^+^ by proton transfer to H_2_O, which also initiates the formation of large cluster ions. At higher humidity content, O is created by reactions between OH radicals, and lost by recombination with OH. OH is produced mainly from H_2_O^+^ by proton transfer to H_2_O and by electron impact dissociation of H_2_O. It is lost by reactions with other OH molecules to form either H_2_O + O or H_2_O_2_. Formation pathways change as a function of humidity content and position in the plasma channel. The understanding of the chemical kinetics of O and OH gained in this work will help in the development of plasma tailoring strategies to optimise their densities in applications.

## Abstract 2

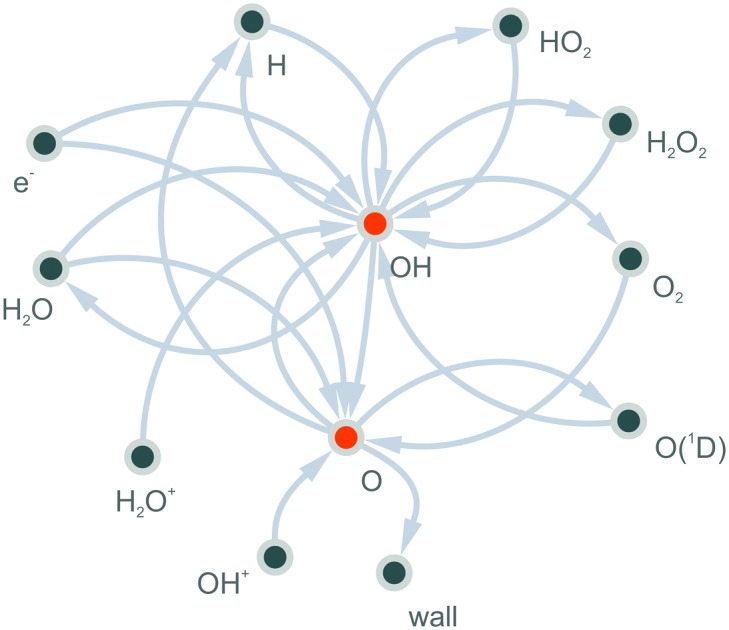
Investigating the formation and kinetics of O and OH in a He–H_2_O plasma jet using absorption spectroscopy and 0D modelling.

## 1 Introduction

The interaction of non-thermal atmospheric pressure plasmas (APPs) with biological matter and their potential applications as biomedical devices^1^–^4^ are currently a topic of significant interest. APPs have been shown to be effective in many different areas of biomedicine, such as sterilization,^5^–^7^ cancer treatment,^8^–^12^ and wound healing,^13^–^15^ and have recently been identified as potential triggers of beneficial immune responses.^16^ First trials on patients confirm the effectiveness of APPs.^13^,^17^,^18^ APPs may offer advantages compared to conventional therapeutics due to their typically small dimensions, offering the possibility of locally confined treatment, low production cost, and the potential to tailor sources for specific applications.

A key question in plasma interactions with biological matter is the role played by plasma produced reactive species (RS). RS are known to interact with cells and their membranes, and often serve as signaling agents in cell metabolism.^19^,^20^ They can also cause severe damage to cells at high concentrations.^19^,^21^

For APPs to fulfil their potential in any biomedical application, a full characterization of the sources used to produce them is necessary, including the quantification of RS produced. Reactive oxygen and nitrogen species (RONS), such as atomic oxygen and nitrogen (O and N), ozone (O_3_), excited states of molecular oxygen (*e.g.* O_2_(a ^1^Δ)), or nitric oxides, have previously been quantified both experimentally and numerically in O_2_ and N_2_ containing plasmas.^22^–^28^ Here, the production of RS in an enclosed APP operating in helium with small contents of humidity is investigated. Water is typically present in the direct vicinity of biological material, and can easily enter the gas phase *via* evaporation. Therefore, RS produced from water vapor can be created during the treatment of the material when plasmas are applied. Water is also usually present as a feed gas impurity.^29^ Therefore, the investigation of RS directly produced from water vapor, such as O and the hydroxyl radical OH, is of interest for biomedical applications. These species can act as precursors for longer-lived species such as hydrogen peroxide (H_2_O_2_), an important signaling agent in cells,^19^,^30^ and O_3_. In high concentrations both of these species can have toxic effects on biological material.

The quantification of RS in APPs represents a challenge for diagnostics based on optical emission from excited states, since the plasma emission is strongly quenched by the ambient gas due to the high pressure. Laser Induced Fluorescence (LIF) and Two-photon Absorption LIF (TALIF) have been previously used to detect species such as O and OH produced from water vapor.^31^–^34^ However, in order to accurately predict the effect of quenching using these techniques at atmospheric pressure, the densities of all potential quenching particles are needed. This is increasingly challenging in complex gas mixtures and in regions with gradual gas mixing, like the plasma effluent. These techniques also rely on quenching rate coefficients for investigated species with all possible quenchers, which for some cases, particularly quenching involving water molecules, are only poorly known. The implementation of faster laser systems such as picosecond or femtosecond lasers^35^–^37^ can help to quantify the effect of these quenching processes. In addition to accounting for the effects of quenching to obtain absolute density measurements using TALIF, an additional calibration measurement involving a gas with a known quantity is typically needed. An alternative diagnostic technique, which is independent of collisional quenching, is mass spectrometry. This technique has recently been used to detect RS such as OH and H_2_O_2_ produced from water vapor in the plasma effluent.^38^ Similar to LIF and TALIF, this technique requires a calibration measurement to obtain absolute species densities. Mass spectrometry has also been used to detect high order protonated water clusters^39^,^40^ produced in APPs. Species deposited in a liquid by plasma treatment are sometimes investigated by means of absorption spectroscopy in the liquid phase, and electron paramagnetic resonance spectroscopy.^41^,^42^ However, to calculate gas-phase densities from liquid-phase densities, usually a calibration is required.

An established optical diagnostic technique for the quantification of OH in the gas-phase is ultra-violet (UV) Absorption Spectroscopy (AS),^34^,^43^–^45^ which is independent of collisional quenching and does not require an additional calibration measurement. However, measuring ground state densities of atomic species produced in water-containing plasmas, such as O, is challenging using AS since the energy gaps between the ground and excited states of the atoms are large. Therefore, the required excitation wavelengths typically lie in the vacuum ultra-violet (VUV) spectral range, which is strongly absorbed by air. However, atomic species, in particular O and N, have previously been quantified in an APP using synchrotron radiation and a spectrometer with an ultra-high spectral resolution, so-called VUV high-resolution Fourier-Transform Absorption Spectroscopy (VUV-FTAS).^22^,^23^

In this work, we combine VUV-FTAS and UV broad-band AS (UV-BBAS) to determine absolute densities of gas-phase O and OH in a radio-frequency APP jet operated in helium (He) for different values of humidity up to 1.3%. We combine the experimental investigations with zero-dimensional, plug-flow plasma simulations to model the chemical kinetics in the source. These models are commonly used to study properties of atmospheric pressure plasmas.^46^–^51^ The role of humidity on the plasma chemistry in APPs has been subject of numerical investigations in the past,^48^,^52^–^54^ that have established the baseline understanding of these systems. We build upon these prior works by comparison of modeling results to experiments performed for the same conditions. Species densities are measured and simulated mainly in the plasma bulk, to validate the reaction mechanism. The resulting reaction mechanism can then be used to investigate important formation pathways for different RS, and to predict additional species densities that are difficult to measure. In many applications, reactive species exit the plasma source into ambient air where the chemical kinetics will differ from the active plasma region. This transition is not investigated here, but the validated reaction mechanism constructed in this work will act as a base to be built upon for future studies in this area.

## 2 Experimental setup

### 2.1 Atmospheric pressure plasma jet

**Fig. 1:**
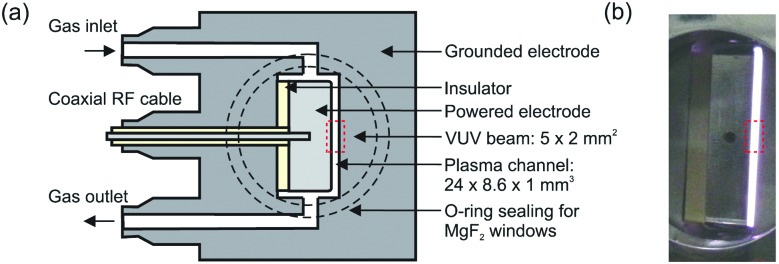
(a) Schematic cross-section and (b) photograph of the plasma source. The perpendicular orientation of the synchrotron vacuum ultraviolet (VUV) beam with respect to the plasma channel is indicated by the dashed rectangle. Images taken from Dedrick *et al.*,^23^ under a Creative Commons Attribution 3.0 licence (; https://creativecommons.org/licenses/by/3.0/).

The production of atomic oxygen (O), and hydroxyl radicals (OH), in an atmospheric pressure plasma jet (APPJ) operating in 5 slm helium (either grade 4.6 with 25 ppm N_2_ and 7 ppm O_2_ impurities, or grade 5 with 3 ppm H_2_O and 2 ppm O_2_ impurities) with water vapor (H_2_O) admixtures is investigated. The plasma jet used in this work is shown in Fig. 1 and is the same as described by Dedrick *et al.*^23^ The jet has a plane-parallel electrode configuration. One electrode with an area of 2.4 × 0.86 cm^2^ is powered by a sinusoidal voltage at a frequency of 13.56 MHz while the other electrode, which is the housing of the source, is grounded. Powered and grounded electrodes are separated by 0.1 cm, keeping the critical dimensions and operating parameters close to the ‘COST Reference Microplasma Jet’,^55^ but with a smaller surface-to-volume ratio (22 cm^–1^ here instead of 40 cm^–1^ for the ‘COST Reference Microplasma Jet’). An impedance matching network unit (L-configuration) is used to optimise the power coupled into the plasma. The applied voltage across the gap is monitored using a high-voltage probe. A list with the equipment for the power coupling and voltage monitoring can be found in Appendix B.

We generally conduct experiments at a fixed generator power. For measurements of OH, we set the generator power close to the arcing point of the plasma in pure helium, which is independent of the generator power when using different generators and which typically occurs around 520 ± 10 V_pp_. Starting at this point also maximises the measurement range with respect to water content. The water content is varied while keeping the settings on the generator constant. The implications of this on the coupled power to the plasma will be further discussed in Section 2.4. At higher voltages, the plasma tends to extend around the powered electrode when operated in pure He, and transitions from a homogeneous glow-like discharge into a constricted “arc” mode at the electrode edges, which can damage the source.

For the O measurements, the source is operated in a vacuum vessel, with limited options for visually identifying the plasma mode through a transparent vacuum flange. In this case measurements are carried out at a lower voltage of 470 V_pp_ to avoid the ‘constricted mode’ and related damage of the electrodes.

For the spatially resolved measurement of OH (presented in Section 4.1), a different plasma source, as described elsewhere,^22^ has been used. It utilises the same design concept, *i.e.* the same gap size of 1 mm, but a slightly larger electrode area of (1.1 × 3) cm^2^, compared to the source described earlier. Since the surface-to-volume ratios for both designs are very similar, we assume the RS production to be comparable under similar operating conditions (gas flow, power density).

Water vapor is admixed into the He flow using two mass flow controllers and a homemade bubbler, which consists of a domed glass adapter (Biallec GmbH) clamped to a KF40 flange. Two stainless steel pipes welded to the flange provide gas in- and out-lets. Both mass flow controllers are fed with dry He, while the outlet flow of one controller passes through the bubbler before being mixed with the other. By changing the ratio of the humidified to the dry He flow, the water vapor content of the total gas flow can be regulated.^34^,^43^,^45^ With the humidity level in the He flow leaving the bubbler being saturated (see below), the vapor pressure 
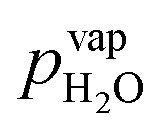
 can be calculated using the semi-empirical expression given below.^56^ The total amount of water in the vapor phase can then be calculated using the vapor pressure of H_2_O (in bar) and the flow rate of the He through the bubbler *F*bubblerHe, as described in ref. 34:1
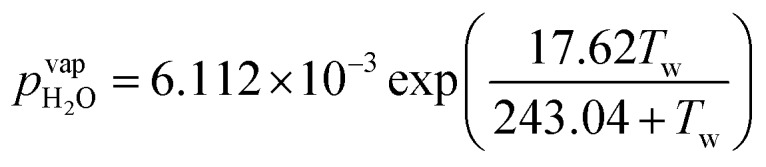

**Fig. 2:**
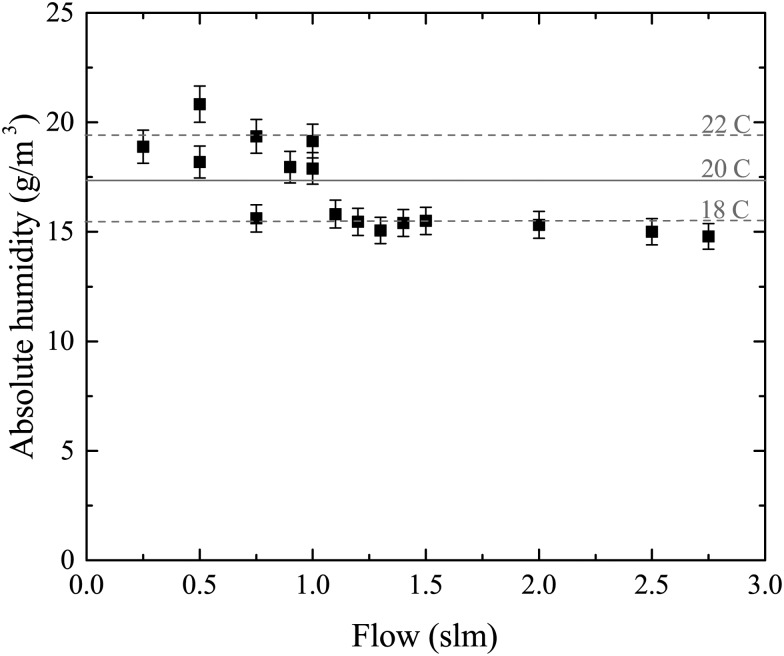
Measured absolute humidity in the gas phase as a function of the He flow through the bubbler. Horizontal lines represent different theoretical values for the water temperature. For calculations of the water content in later experiments a temperature of (20 ± 2) °C is assumed, as indicated as solid and dashed lines.

2
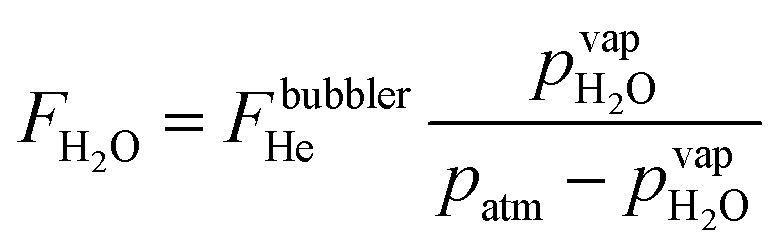
where *T*_w_ is the water temperature in °C. To check that the He flow exiting the bubbler is saturated with H_2_O, the weight loss of the bubbler due to evaporation of the water is measured for different He flow rates as a function of time. The absolute water concentration in the He flow is given by the ratio of the two former quantities. The results are shown in Fig. 2. A small systematic drop of the measured water concentration with increasing He flow is observed. This may reflect a temperature drop of the water inside the bubbler, which is not temperature controlled, either because of an increased evaporation rate, or fluctuations in the laboratory room temperature, since measurements were taken over several days. The averaged result for the water concentration of (16.9 ± 2.0) g m^–3^ corresponds to a water temperature of (20 ± 2.0) °C assuming full saturation. Since this temperature represents the typical ‘room temperature’ at which the measurements were taken, we can confirm that the He flow out of the bubbler is saturated with water vapor. The uncertainty of 2 °C would lead to an uncertainty of approximately 14% in the calculation of the water vapor content in the gas.

### 2.2 VUV high-resolution Fourier-transform absorption spectroscopy

Absolute line-averaged O atom ground state densities are measured at the DESIRS beamline at the synchrotron SOLEIL,^57^ with its unique ultra-high resolution VUV Fourier-Transform spectrometer^58^ able to cover the complete VUV spectral range down to 40 nm with a resolving power (*λ*/Δ*λ*) of up to 10^6^. The atomic oxygen transition O(2p^4 3^P_*J*=2_ → 3s ^3^S_1_) is investigated in this work.

The measurement and analysis procedure is described in detail elsewhere.^22^ The spectrometer yields a transmission spectrum *S*_T_, which includes the convolution of the plasma transmission *T* (accounting for Doppler and pressure broadening of the corresponding spectral line profile) with the sinc-shaped instrumental function 
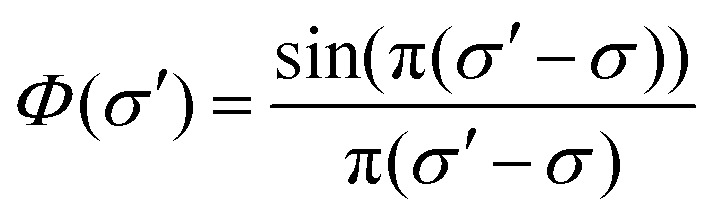
 of the FT spectrometer

3 *S*_T_(*σ*′) = *S*_0_(*σ*′)[*Φ*(*σ*′ – *σ*) × *T*(*σ*)].

where *σ* is the wavenumber of the transition and *S*_0_ the reference spectrum without absorber. Absolute densities are obtained from the transmission spectra using Beer–Lambert's law

4 *T*(*σ*) = exp(–*A*(*σ*)) = exp(–*k*(*σ*)·*l*),

where *A*(*σ*) is the absorbance, *l* the length of the absorbing medium (here defined by the width of the electrode as 8.6 mm), and *k* the absorption coefficient, which includes the ground state density, the spectral line “Voigt” profile, which takes into account the Doppler and pressure broadening of the spectral line, the statistical weights *g*_*J*_ for the different states and the transition probabilities. For the evaluation of these transmission spectra, the different broadening mechanisms are taken into account as fixed values during the fitting process. The instrumental broadening is set to Δ*σ*_I_ = 0.87 cm^–1^ as described elsewhere.^22^ The Doppler width Δ*σ*_D_ = 0.24 cm^–1^ is calculated for a gas temperature *T*_g_ = 304 K. This value is determined as the OH rotational temperature from the absorption measurements by fitting the OH(X ^2^Π_*i*_, *υ*′ = 0) → OH(A ^2^Σ^+^, *υ*′′ = 0) rotational transitions using a spectral simulation (see results discussed in Section 4.2 and shown in Fig. 9). The detailed working principle of the simulation will be described in the next section. Rotational temperatures were obtained for different water contents ranging from 0.1 to 1.3%. A standard deviation of 2.2 K shows that the gas temperature stays fairly constant within the investigated range of H_2_O admixtures. Finally, the pressure broadening is determined as Δ*σ*_L_ = 0.37 cm^–1^ from an average of several automated fits to the data using the previously specified values for Δ*σ*_D_ and Δ*σ*_I_. This value is in reasonable agreement with Δ*σ*_L_ = (0.46 ± 0.03) cm^–1^ for He measured by Marinov *et al.*^59^ in the ‘COST Reference Microplasma Jet’ using Doppler-free TALIF, albeit for a different optical transition.

**Fig. 3:**
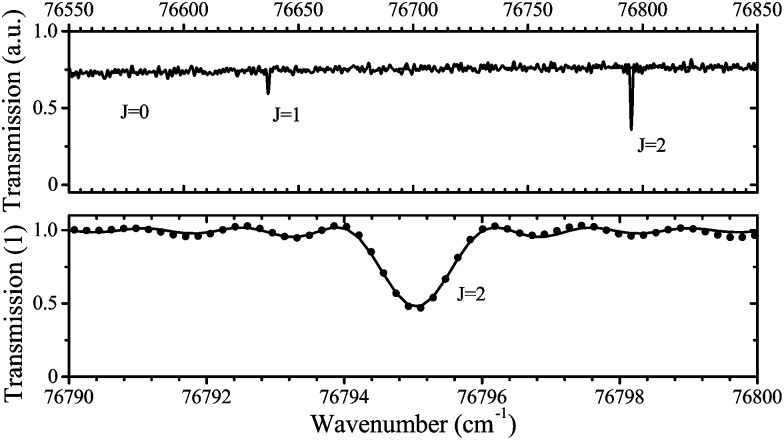
Example measured (points) and fitted spectrum (solid line) for the measured transition O(2p^4 3^P_*J*_) → O(3s ^3^S_1_) using VUV-FTAS. Top: Broad spectral range. The transition from the ground *J* = 0 sub-level is at *σ* = 76 568 cm^–1^ (not visible). Bottom: Zoom into the transition from the *J* = 2 sub-level of the ground state. The spectrum was taken with 4.6 slm dry He and 0.4 slm humidified He, equivalent to 1880 ppm of water vapor in the gas phase. The O density obtained from this spectrum is 2.7 × 10^13^ cm^–3^.

A typical transmission spectrum is presented in Fig. 3. We only evaluate the strongest *J* = 2 transition of the O(2p^4 3^P_*J*=2_) triplet from the fine structure split ground state to the first electronically excited state because of the low signal-to-noise ratio of the weaker *J* = 0, 1 transitions. In order to estimate the total ground state density 
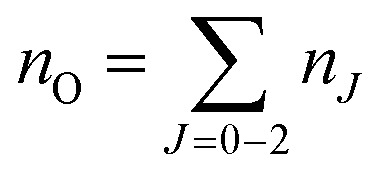
, the Boltzmann factor5
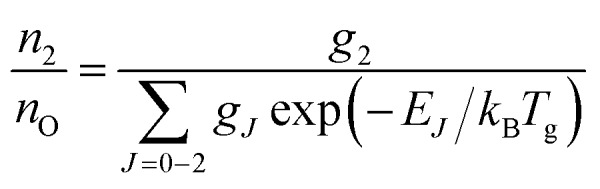
is applied, where *E*_*J*_ is the energy of the state and *k*_B_ is the Boltzmann constant.

The main uncertainties in this technique lie in the estimated absorption length (uncertainty of 5%), and the accuracy of the transition probability *A*_*ik*_ (≤3%^60^), which are included in the expression for the absorption coefficient in eqn (4). A change of the gas temperature within 10 K influences the Boltzmann factor calculated using eqn (5) by less than 2%. We therefore estimate the systematic error in all VUV-FTAS measurements presented here to be within 10%.

### 2.3 UV broad-band absorption spectroscopy

**Fig. 4:**
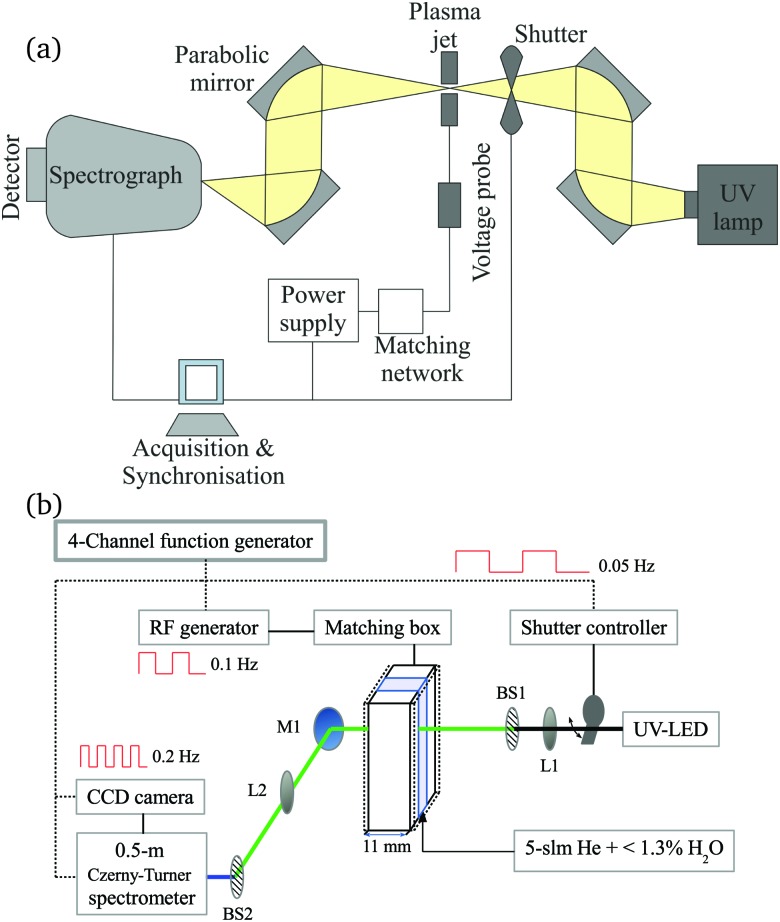
Schematics of the experimental setups used for the UV-BBAS measurements of OH. UV-BBAS I (a) and UV-BBAS II (b). Figure (b) was adapted from Wijaikhum *et al.*,^24^ under a Creative Commons Attribution 3.0 licence (; https://creativecommons.org/licenses/by/3.0/).

Absolute OH densities are measured in the same plasma source using UV-BBAS using two different experimental setups to ensure reproducibility. The experimental setup UV-BBAS I is presented in Fig. 4(a). Light from an ultra-stable broad-band plasma lamp (Energetiq EQ-99) is guided through the middle of the plasma channel and focused on the entrance slit of a 320 mm spectrograph (Isoplane SCT320) with a 2400 grooves per mm grating. Spectra are recorded using a photodiode array detector (Hamamatsu S-3904). The setup is described in detail elsewhere.^61^

The second setup (UV-BBAS II), which is shown in Fig. 4(b), comprises several different components, mainly a UV LED (UVTOP-305-FW-TO18, Roithner Lasertechnik GmbH) as light source and a CCD camera (Andor Newton 940) in combination with a spectrometer (Andor SR-500i) as detector. For the UV-BBAS II setup, the plasma is mounted on an automated *x*–*z* stage, allowing for spatially resolved measurements in the plasma channel. The experimental setup is described in detail elsewhere.^24^

**Fig. 5:**
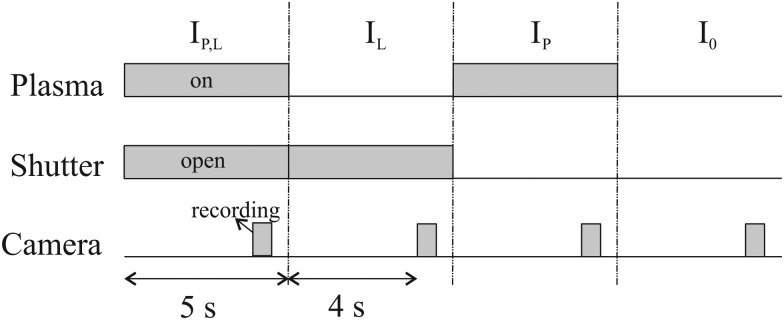
Trigger scheme for all UV-BBAS absorption measurements.

To calculate the absorbance in eqn (4), four signals are required: plasma on and light source on (*I*_P,L_), plasma on only (*I*_P_), light source on only (*I*_L_) and a background with both plasma and light source off (*I*_0_). Each signal is integrated over a time period of 50 ms, with a plasma stabilisation time of 4 s beforehand. A schematic showing this sequence is shown in Fig. 5. The plasma transmission *T* in eqn (4) is calculated as6
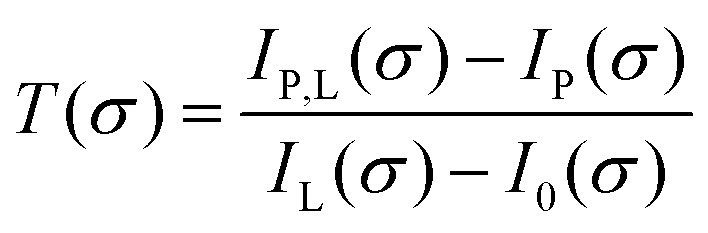



**Fig. 6:**
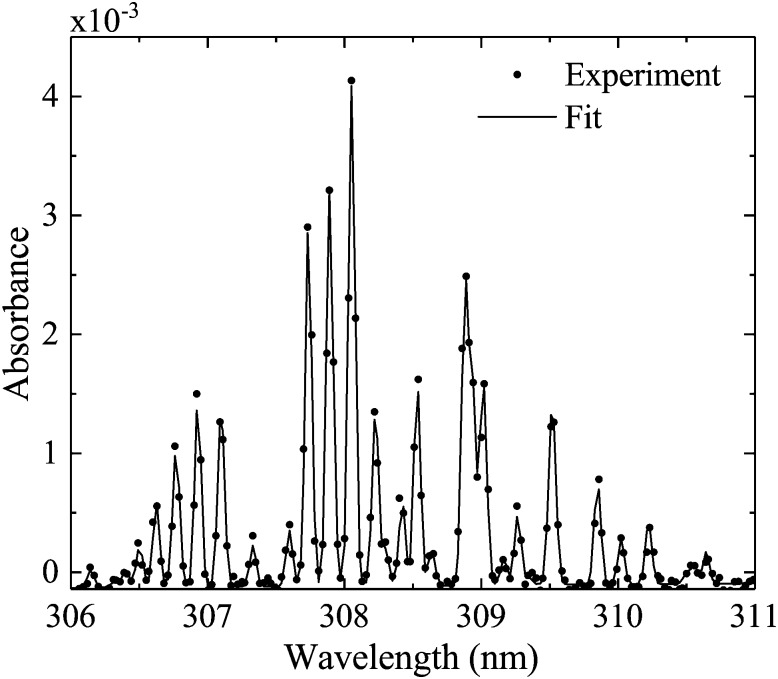
Example spectrum for the measured and fitted transition OH(X ^2^Π_*J*_, *υ*′ = 0) → OH(A ^2^Σ+*J*, *υ*′′ = 0) using the UV-BBAS I setup, for 4 slm dry and 1 slm humidified He, 530 V_pp_. The calculated OH(X ^2^Π_*J*_, *υ*′ = 0) density obtained from this spectrum is 2.6 × 10^14^ cm^–3^.

An example spectrum of the OH absorbance *A* is shown in Fig. 6. Using two setups, an admixture range of 200–13 000 ppm humidity content is investigated.

Measured OH rotational absorbance spectra of the transition OH(X ^2^Π_*i*_, *υ*′ = 0) → OH(A ^2^Σ^+^, *υ*′′ = 0) are fitted using a spectral simulation in order to obtain absolute OH(X ^2^Π_*i*_, *υ*′ = 0) densities. The fitting programme is based on a calculation of the Einstein coefficients and wavelengths for the individual transitions within the investigated rotation band, as described by Dieke and Crosswhite.^62^ Based on the selection rules for the total angular momentum *J* = *L* ± *S* and the angular momentum *L* (without electron spin *S* = 1/2 for the OH radical), relative intensities are calculated for 12 possible branches, using expressions derived by Earls.^63^ An experimental value for the radiative lifetime for a rotationless upper state *F*_1_(*J*′′ = 0.5) has been determined as 0.688 μs^64^ (here, *F*_1_ donates the doublet component of the upper state with *J* = *L* + 1/2, in accordance with Diecke and Crosswhite^62^). Therefore, all calculated relative Einstein coefficients can be normalised to this value. Our calculated values are in good agreement with those from Goldman and Gillis.^65^ As in Dilecce *et al.*,^45^ the spectral fitting includes an instrumental function, whose width represents the spectral resolution of the spectrometer, which depends on the pixel size of the detector array, the optical grating and the width of the spectrograph's entrance slit. We assume the instrumental function to be Gaussian. Examples of measured and simulated absorbance spectra are shown in Fig. 6. Here, the instrumental width is 56 pm (UV-BBAS I) or 34 pm (UV-BBAS II), which is much larger than the Doppler (Δ*λ*_D_ (304 K) = 0.098 cm^–1^ = 0.93 pm) and pressure broadening (estimated as Δ*λ*_P_ (1 atm) = 0.07 cm^–1^ = 0.66 pm, as in ref. 66). The fitting programme is also used to calculate OH rotational temperatures.

The main systematic uncertainties of UV-BBAS lie in the estimation of the absorption length (5%), and the accuracy of the calculated Einstein coefficients, which we estimate here to be within 10%. For the absorbance measured with the UV-BBAS II setup (featuring the LED), the standard deviation of the noise is in the order of 3 × 10^–4^, which places a lower limit on the measurable OH density at 3.6 × 10^13^ cm^–3^. For the UV-BBAS I setup (featuring the ultra-stable light source), the noise level of the measured absorbance is typically an order of magnitude lower, and therefore disregarded in the uncertainty estimation. The combination of the systematic error and a statistical error of 7% is shown as error bars in the results that follow.

### 2.4 Determination of plasma power

For accurate comparison between simulation and experiment the rf power dissipated in the plasma is a particularly important. Experimentally, this so-called plasma power is measured by determining current, voltage and phase shift using current (Ion Physics Corp. CM-100-L 1 V/A) and voltage probes (PMK-14KVAC). The probes are installed between the impedance matching unit and the plasma source. The time averaged power *P* is given by7
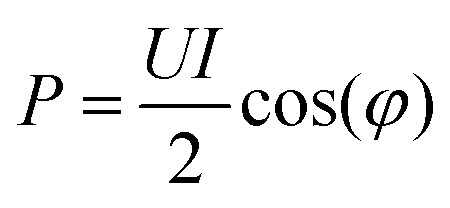
where *U* and *I* are the voltage and current amplitudes, respectively, and *φ* is the phase shift between the two. Parasitic power losses, *e.g.* into the plasma source or the rf cables, are accounted for by measuring the power deposited in the system without a gas flow, so that the ignition of the plasma is inhibited. The subtraction method is then used^67^,^68^ for a given current to determine the plasma power

8 *P*_d_(*I*^2^) = *P*_on_(*I*^2^) – *P*_off_(*I*^2^)

The net power *P*_d_ is the difference between the power measured with and without plasma, *P*_on_ and *P*_off_, respectively. For a given plasma volume *V*_plasma_, the corresponding plasma power per unit volume *p*_d_ is given by9
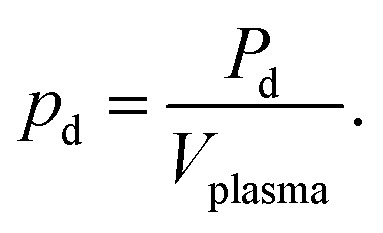



The instrumental phase shift of the measurement system (probes, BNC signaling cables, and digital oscilloscope) is determined using a variable air capacitor with known phase shift (MFJ 282-2018-1). For the calibration measurement, the plasma source with its rf cable to the matching box is replaced by this capacitor.

Current and voltage waveforms are recorded by a fast oscilloscope (LeCroy WaveSurfer10, 10 GS per s sample rate). The voltage and current amplitude as well as the corresponding phase shift are determined by a Fourier analysis of this data. *P*_d_ was found to be approximately constant (within 15%) as a function of feed gas water content at a constant generator power and matching settings. The average of *P*_d_ over several different water contents is used as an input for the simulations over the whole range of water content. The average value of *P*_d_ was determined as 2.8 W (≈14 W cm^–3^) for the UV-BBAS measurements of OH (at approximately 510 V_pp_), and 2.1 W (≈10 W cm^–3^) for the measurement of O using VUV-FTAS (at approximately 470 V_pp_). These values are used as the input for the simulations, unless otherwise stated.

Power measurements are carried out separately from the density measurements using two power generators: coaxial RFG-150-13 (150 W maximum output power, same model as used for the OH measurements using the UV-BBAS II setup), and coaxial RFG-50-13 (50 W maximum output power, smaller range of powers for a better stability). We find a similar average power using these two setups, and that the power stays constant as a function of water content within one measurement set with a standard deviation of all points below 5%. We estimate a total uncertainty of 15% from repetitive measurements. These variations are small enough to not significantly influence measured species densities, particularly for OH, which we found to be only weakly dependent on applied voltage, and therefore power (increase of about 40% when the voltage is increased from 490 to 850 V_pp_, not shown here).

## 3 Plasma simulations

### 3.1 Model description and reaction mechanism

To better understand the dynamics of reactive species in cold atmospheric pressure plasmas, zero-dimensional plasma chemical kinetics simulations (global models) are often used.^51^ In this work, experimental results are compared to those obtained using the GlobalKin code as described elsewhere.^50^ GlobalKin solves the continuity equation for mass conservation for both charged and neutral species, taking into account particle production and loss through gas phase reactions and interactions with surfaces10
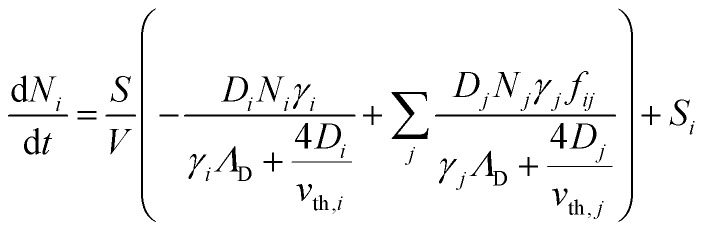
where *N* denotes the number density of heavy particles *i*, 
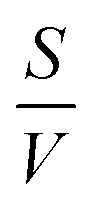
 the surface to volume ratio, *Λ*_D_ the diffusion length, *D* the diffusion coefficient, *γ* the surface sticking coefficient, *f* the return fraction of species from walls, and *S*_*i*_ the source term, which accounts for gas phase production and losses. In addition, the electron energy conservation equation is solved to calculate the electron temperature in the plasma by taking into account the balance of power input and loss of electron energy due to elastic and inelastic collisions with heavy particles11
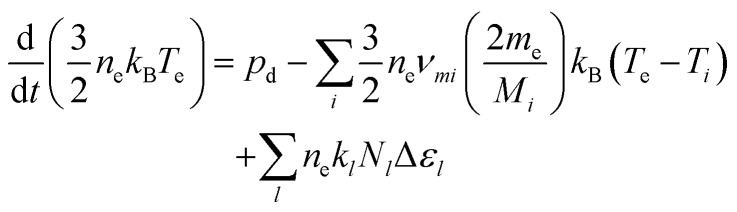
where *n*_e_ is the number density of electrons, *T*_e_ the electron temperature, *m*_e_ and *M*_*i*_ the electron and heavy particle masses, respectively, *ν*_*mi*_ the electron collision frequency, *k* the reaction rate coefficient and Δ*ε*_*l*_ the electron energy gain/loss through inelastic collisions. GlobalKin also incorporates a two-term approximation Boltzmann solver, which updates the electron energy distribution function during the simulation, and calculates electron impact rate coefficients, using electron impact cross sections as an input. From the electron energy distribution function electron transport coefficients are also determined for the use in the continuity equation.

In this work we apply a temporally constant power deposition corresponding to the time averaged power measured in the experiment. For rf APPs the electron heating is strongly modulated in time, leading to a power and electron impact rate coefficients, that vary during the rf cycle.^69^,^70^ This effect is not captured in our model. However, Lazzaroni *et al.*,^70^ investigated the differences between a conventional global model, using a time averaged power deposition, and one that takes into account time-varying power deposition within the rf period. For their case, using a He/O_2_ reaction mechanism, the densities of neutral species calculated by the modified model (O, O_3_, O*, O_2_*) were typically within a factor of 2 of those from the conventional model. The trends in the results of the two models were similar. Therefore, we expect that neglecting the time-varying power deposition in our model will only lead to a quantitative difference in the results, while the trends should remain valid.

Gas temperatures are self-consistently calculated using the GlobalKin code using^50^12
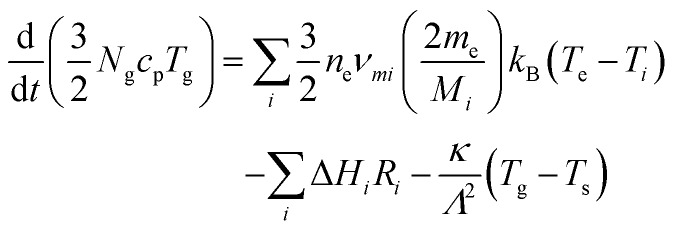
Here, *N*_g_ is the gas density, *c*_p_ the specific heat of the gas, *T*_g_ the gas temperature, Δ*H*_*i*_ the change of enthalpy for reactions with rate *R*_*i*_, *κ* the thermal conductivity of the gas, and *T*_s_ the surface temperature of the reactor wall. Therefore, GlobalKin balances gas heating *via* electron collisions (first term on right hand side), chemical reactions (second term), and heat exchange with surrounding walls (third term). Here, we assume *T*0g = 295 K (room temperature) as the initial temperature of the gas before entering the plasma channel. Coupled powers are typically small in this work, therefore it is assumed that the reactor wall is not significantly heated and *T*_s_ is set to 295 K. This is in good agreement with previous observations,^24^ where the electrode temperature was measured using an infrared thermometer in a very similar plasma configuration under a variation of plasma power.

The model incorporates 43 species and 390 reactions. Table 1 contains the species in the mechanism. The plasma reaction mechanism is in Appendix A (Tables 5–8). At the surfaces, it is assumed that most neutral and negatively charged species (except electrons) do not react, while positive ions are neutralised with a probability of 1. The species assumed to react differently are listed in Table 4 in the Appendix A. A detailed discussion of the role of surface interactions in a similar simulation system is given elsewhere.^71^

**Table 1:** Species considered in the simulation

	Neutral	Positive	Negative
He	He, He*, He_2_*	He^+^, He_2_^+^	
O	O, O(^1^D), O(^1^S), O_2_, O_2_(a ^1^Δ), O_2_(b ^1^Σ), O_3_	O^+^, O_2_^+^, O_4_^+^	O^–^, O_2_^–^
H	H, H_2_		H^–^
OH	OH, HO_2_, H_2_O, H_2_O_2_	OH^+^, H_2_O^+^(H_2_O)_*n*=0,1_	OH^–^, H_2_O_2_^–^, OH^–^(H_2_O)_*n*=1–3_
		H^+^(H_2_O)_*n*=1–9_	
		O_2_^+^(H_2_O)	
Others			e

In this work, the model is solved for a channel length of 2.4 cm, with a gas flow rate of 5 slm, which corresponds to a gas velocity of about 11 m s^–1^. Using a pseudo-1D plug flow, temporally computed densities are converted into spatially dependent quantities. Where species densities are presented as a function of humidity content, densities are extracted from the simulation at the axial centre of the source (at 1.2 cm), which is the position where measurements were made.

From the plasma dimensions, the diffusion length *Λ*_D_, a necessary parameter for determining diffusion losses of particles, is calculated as^72^13
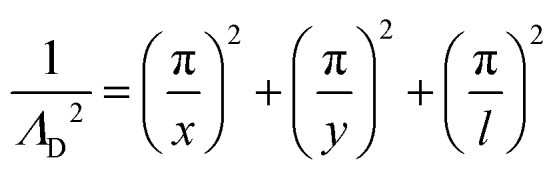
for a plasma with rectangular cross section (*x* × *y*) and length *l*. For the plasma source used here, *Λ*_D_ = 0.0316 cm. (This is larger than the ‘COST Reference Microplasma Jet’ with *Λ*_D_ = 0.0225 cm.)

### 3.2 Pathway analysis

The PumpKin software^73^ is used to identify the production and destruction pathways for the selected neutral species. The reaction pathway of a species of interest results from (a) analysing the elementary reactions that contribute directly or in subsequence to the formation of this species and selecting the significant ones only, (b) algebraically summing up the formal notations of these reactions, and (c) eliminating shorter-lived species, here the electrons and some ions, to end up with a simplified net reaction. The short-lived species are defined as those with a lifetime shorter than a lifetime set by the user, which we denote as *τ*_p_. Note, that this so-called net reaction should not be mistaken as an elementary reaction, since it is specific to the choice of eliminated species. This approach is particularly useful for understanding the production and destruction of species which are formed *via* complex reaction pathways involving a chain of elementary reactions, as opposed to simply one or two.

Among the neutral species considered in this work He* and He_2_* metastables have the shortest effective lifetime. For the pathway analysis, we therefore choose *τ*_p_ to be slightly shorter than the lifetime of these species, which is in accordance with previous work.^52^Table 2 shows the strong dependence of the simulated lifetime on the humidity admixture for specific plasma conditions. These findings support the conclusion of Niemi *et al.*,^46^ that the metastable character of these helium species at high pressure is significantly reduced in the presence of small admixtures or even impurity levels of molecular gases through Penning ionization under atmospheric pressure conditions.

**Table 2:** Lifetimes of the shortest-lived neutral species calculated by PumpKin for 5 slm He flow and 14 W cm^–3^ plasma power, and varying humidity content. Conditions are the same as for the investigations of OH densities under a variation of the humidity content discussed later in Section 4.2. These lifetimes serve as timescale of interest for the pathway analysis

H_2_O content (ppm)	He* lifetime (μs)	He_2_* lifetime (μs)
10	2.539	1.931
100	0.350	0.188
1000	0.036	0.021
10 000	0.004	0.013

## 4 Results

### 4.1 OH densities along plasma channel

**Fig. 7:**
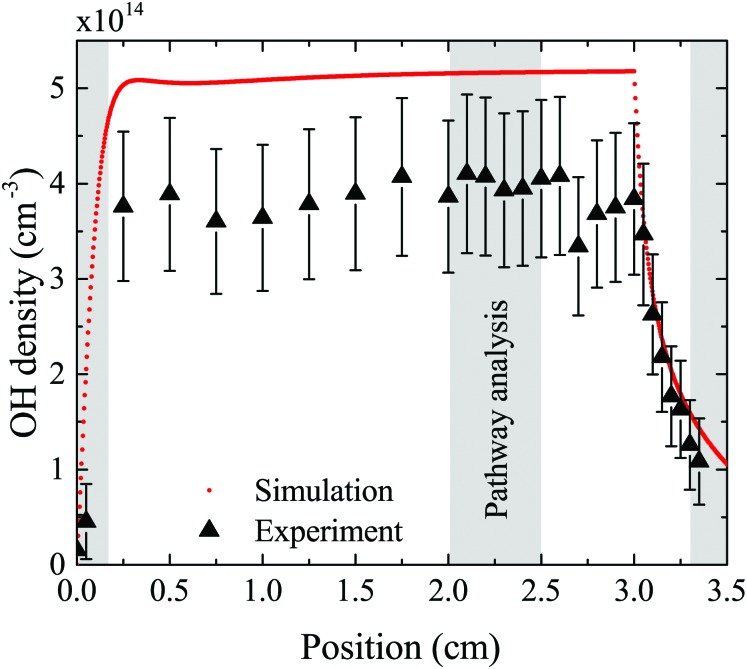
Absolute density of OH as a function of position along the discharge channel, where 0 cm corresponds to the inlet and 3 cm to the outlet of the channel. Experimental results obtained using the UV-BBAS II setup (black triangles) are taken at 18 W cm^–3^ plasma power density, 5 slm total He flow, and 5400 ppm humidity. Corresponding simulation results are indicated by red dots. Shaded areas are used for the PumpKin pathway analysis.

The density of OH along the plasma channel for an intermediate humidity content of 5400 ppm and a plasma power density of 18 W cm^–3^ is shown in Fig. 7. Experimental results show a rapid increase of the OH density over the first 2 mm of the channel. With increasing distance from the gas inlet the density stays approximately constant between 3.5 × 10^14^ cm^3^ and 4.0 × 10^14^ cm^3^ up to the end of the channel. A similar trend is also observed in the simulation. Absolute simulated and experimental densities agree well within around 25%, which is likely within the combined uncertainties of experimental data (as shown as error bars in Fig. 7) and simulations. Uncertainties in the simulation results would most likely occur due to uncertainties in used reaction rate coefficients, and considered reaction pathways, and were shown to be within a factor 10 for a He/O_2_ reaction mechanism under similar plasma conditions.^74^

**Fig. 8:**
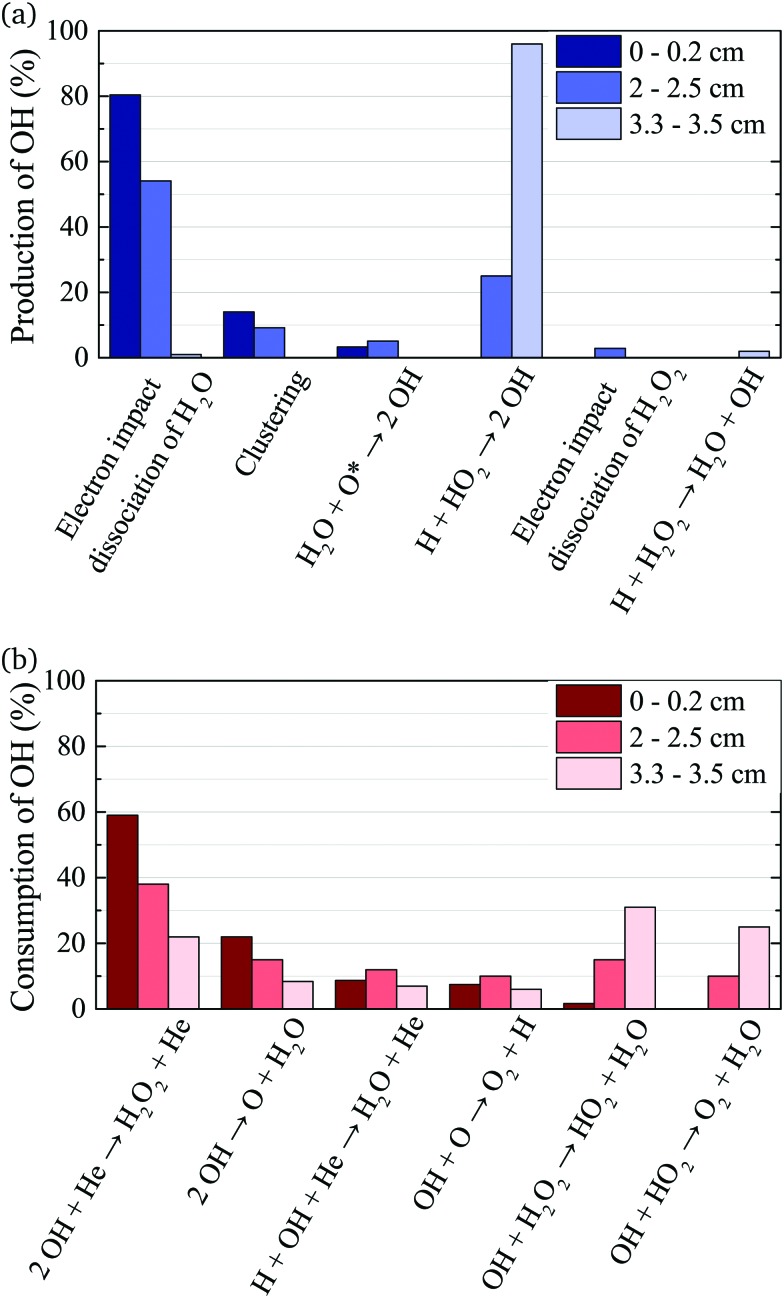
Production (a) and consumption (b) pathways of OH at different positions in the plasma source, as indicated in Fig. 7.

To gain insight into the dynamics of OH formation, a pathway analysis is performed for the three regions highlighted in Fig. 7, which correspond to the fast build-up of OH at the entrance of the plasma channel (0–0.2 cm), a steady-state region (2–2.5 cm) and the decay of OH in the plasma effluent (3.3–3.5 cm). The dominant production and consumption pathways for OH, averaged over each region, are shown in Fig. 8.

At the entrance of the discharge channel (0–0.2 cm), the gas consists mainly of the initial feed gas mixture plus some rapidly forming species such as ions and electrons. Therefore, the main production reaction for OH is through electron impact with water vapor, either *via* dissociation or dissociative attachment

14 e + H_2_O → **OH** + H + e (60%)

15 e + H_2_O → **OH** + H + e (9%)

16 e + H_2_O → **OH** + H^–^ (11%)

The products of eqn (15) are ground state atomic hydrogen and OH in its excited OH(A) state, while the products of eqn (14) are both in their ground states. The percentage contribution for each reaction to the total production of OH is shown in brackets.

Another production mechanism for OH is through the formation, and subsequent destruction, of charged water clusters, as was previously identified by Ding and Lieberman.^52^ The formation of these clusters is typically a multi-step process. For positive clusters, this process usually begins through ionisation of H_2_O, either through electron impact or Penning ionisation with He*. These water ions then collide with water molecules to form the cluster ion H^+^(H_2_O), which accumulates additional water molecules through a series of reactions:17
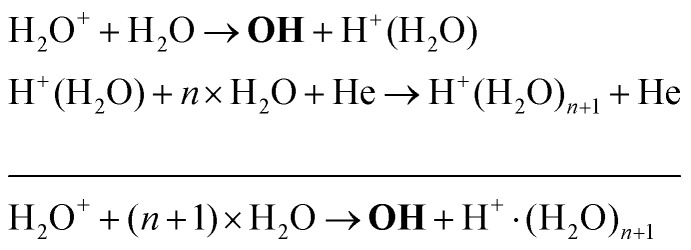
Here, the reaction below the solid line represents the net reaction. Similar processes occur for negative ion clusters OH^–^(H_2_O)_*n*_, which are included in the reaction mechanism for *n* ≤ 3.

The main consumption pathway for OH in the first 0.2 cm of the jet is the formation of H_2_O_2_, and recombination to water through

18 2**OH** + He → H_2_O_2_ + He (59%)

19 2**OH** → H_2_O + O (22%)

20 H + **OH** + He → H_2_O + He (9%)

The rapid increase of OH density within the first two millimeters raises the question, if averaging the pathways over this region is a valid analysis. For both the production and consumption pathways, the contribution of each reaction does not change significantly, if evaluated for separate points within the first 0.2 cm instead of averaging over this region. However, the ratio of the total rate of production and consumption changes significantly, leading to the increase in OH density over this region. Further from the gas inlet, the rates of production and consumption equalise leading to an equilibrium OH density.

In the quasi steady state region (2–2.5 cm), the previous pathways still dominate. However, additional species with intermediate lifetimes build up along the channel and begin to play a role in the formation of OH. For example, hydroperoxy radicals (HO_2_) promote production of OH through reactions with H

21 H + HO_2_ → 2**OH** (25%)

while H_2_O_2_ and HO_2_ lead to the destruction of OH through

22 **OH** + H_2_O_2_ → HO_2_ + H_2_O (15%)

23 **OH** + HO_2_ → O_2_ + H_2_O (10%)

In the afterglow region (3.3–3.5 cm), a rapid decay of OH occurs both in experiment and simulation, as shown in Fig. 7. Short lived species such as ions and electrons recombine rapidly, while metastable species like He* and He_2_* are consumed through Penning ionization with water before reaching this region. Therefore, the chemistry in the plasma effluent is dominated by intermediate and long lived neutral species, where OH is produced mainly through reactions between H and longer-lived neutral species:

24 H + HO_2_ → 2**OH** (96%)

25 H + H_2_O_2_ → **OH** + H_2_O (2%)

In this region, consumption occurs at a higher rate than production, leading to a decrease in the OH density, with reactions (22) and (18) (collisions with H_2_O_2_ and OH) dominating.

### 4.2 OH densities under varying humidity content

**Fig. 9:**
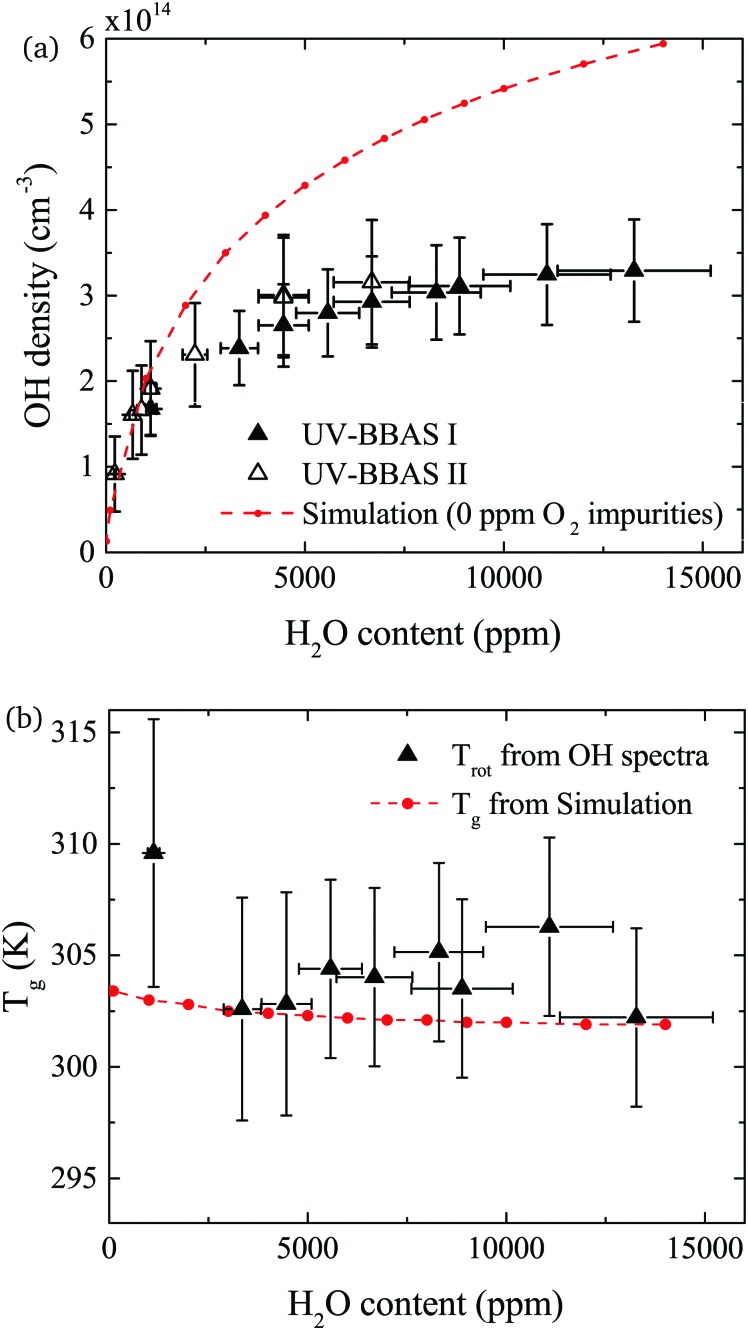
(a) Absolute OH densities as a function of the water content of the He feed gas for a total He flow of 5 slm and 14 W cm^–3^ plasma power. Triangles represent the experimental values (taken in the centre of the plasma channel at *x* = 1.2 cm) and the dashed red line the simulation results. OH densities are measured using two different UV-BBAS setups, as described in the text. (b) Rotational temperatures obtained from the experimental OH(X) absorbance spectra measured with the UV-BBAS I setup (black triangles) and gas temperatures calculated using GlobalKin (red dashed line). Error bars for temperature measurements represent uncertainties resulting from the fitting procedure.

The density of OH measured by UV-BBAS in the centre of the plasma channel (at position 1.2 cm) as a function of the H_2_O content in the feed gas is shown in Fig. 9(a). The OH density increases sub-linearly with increasing H_2_O content, as previously observed.^34^,^43^ Absolute densities obtained in this work also agree well with results obtained by others.^34^,^43^ Absolute OH densities measured with the two different experimental setups agree well within the uncertainties in each measurement.

The simulated OH densities at different feed gas humidity contents are shown in Fig. 9(a). In general, good agreement in the trends of experimental and simulation results is observed. Absolute OH densities agree particularly well at low H_2_O contents <2000 ppm. Towards higher H_2_O contents, simulated densities are higher than those measured experimentally. The largest difference is a factor 1.8 at the highest H_2_O content, which is reasonable agreement given the previously mentioned uncertainties.

OH(X) rotational temperatures and gas temperatures calculated using GlobalKin are shown in Fig. 9(b), and found to be in good quantitative agreement with each other. In both experiment and simulations, temperatures stay fairly constant with increasing water content. While in the simulation, a very small decrease of the gas temperature is observed, the experimental data is more scattered, and a clear trend cannot be observed taking into account the uncertainties of the measurement.

**Fig. 10:**
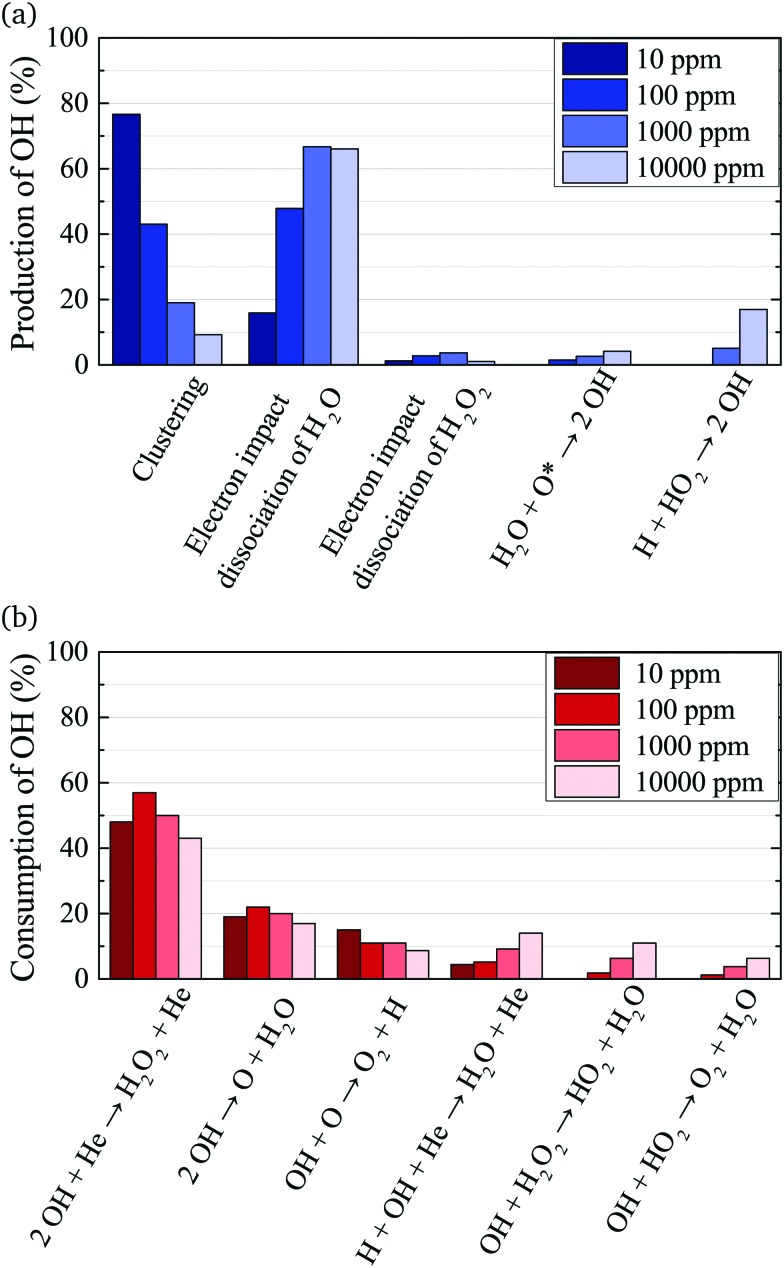
Dominant production (a) and consumption (b) pathways of OH for different H_2_O feed gas contents. The rates from which percentages are calculated are averaged over the whole discharge channel (0–2.4 cm, without effluent region).

The main production and consumption pathways for OH at different H_2_O admixtures are shown in Fig. 10. Two production pathways dominate for all H_2_O admixtures. The dominant pathway for OH production at lower H_2_O contents is *via* Penning ionisation of H_2_O, and subsequent cluster association reactions (eqn (17)). At any stage of the clustering process, the clusters can be destroyed by dissociative recombination with electrons

26 e + H^+^(H_2_O)_*n*_ → H + *n* × H_2_O

Towards higher H_2_O contents, this pathway is gradually replaced by direct electron impact dissociation or dissociative electron attachment with H_2_O (eqn (14) and (16)).

OH is mainly consumed by reactions with other OH radicals (eqn (18) and (19)) and O

27 **OH** + O → O_2_ + H

Towards higher water admixtures, the contributions of these reactions to the consumption of OH decrease slightly, and reactions of OH with H, and more slowly forming species such as H_2_O_2_ and HO_2_ become more important.

**Fig. 11:**
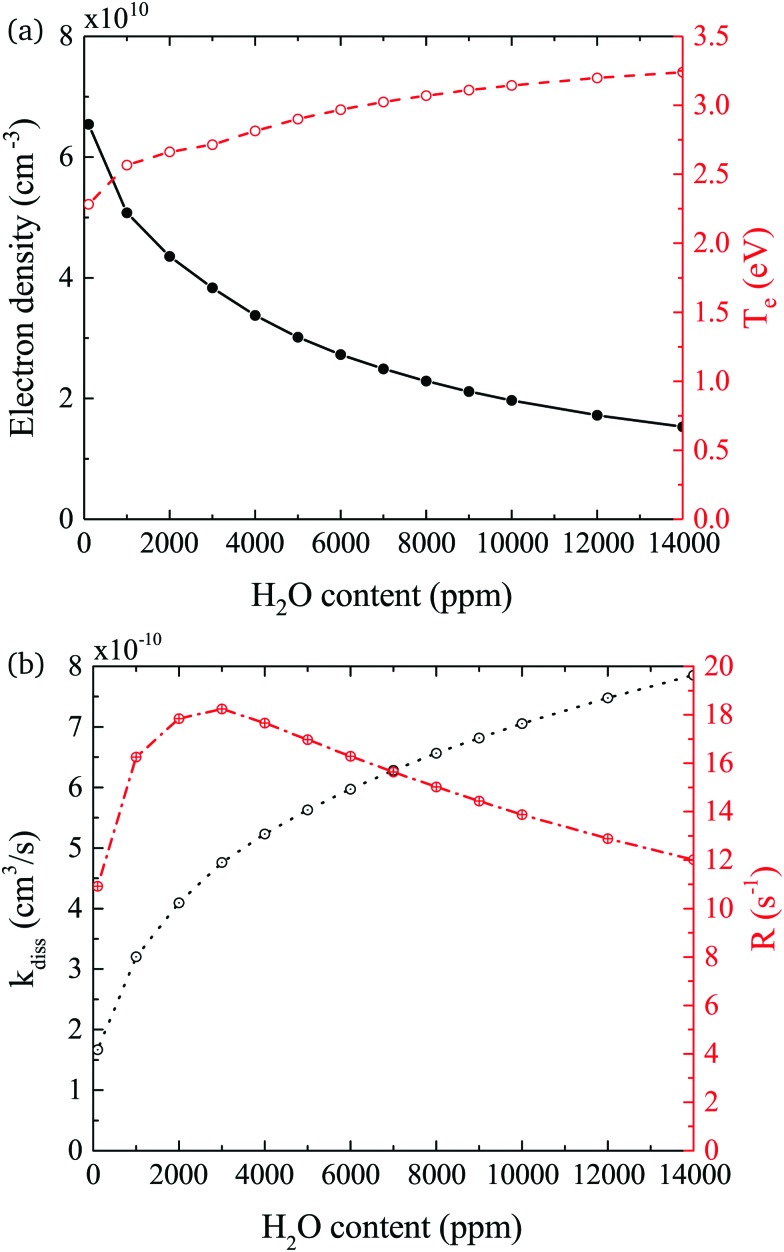
(a) Electron density *n*_e_ and temperature *T*_e_, and (b) combined rate coefficient *k*_diss_ for electron impact dissociation and dissociative attachment and dissociation frequency *R* = *k*_diss_*n*_e_. Conditions are 5 slm total He flow and 14 W cm^–3^ plasma power.

In both experiment and simulation, OH densities increase rapidly with increasing H_2_O at low H_2_O content, and less rapidly at high H_2_O content. The transition between these two regimes occurs at lower H_2_O content (around 2000 ppm) in the experiment compared to the simulation (around 3000 ppm). This leads to the increasing discrepancy between simulation and experiment at higher H_2_O contents where the experimental OH densities saturate and the simulated OH densities continue to slowly increase. The reason for this transition is investigated by looking at the most important formation pathways for OH, which are production by electron impact dissociation and dissociative attachment of H_2_O (eqn (14)–(16)), and consumption *via* reactions with OH to form H_2_O_2_ and H_2_O (eqn (18) and (19)). As the gas temperature remains relatively constant with changing water content, rate coefficients for consumption of OH also stay approximately constant. The reaction rate coefficient for production of OH is dependent on the electron temperature *T*_e_, and the electron density *n*_e_. Fig. 11(a) shows the two quantities as a function of humidity content. *T*_e_ and *n*_e_ show opposite trends with increasing H_2_O admixture. *T*_e_, which is calculated from balancing the electron energy sources and losses (see eqn (11)), increases with increasing H_2_O content due to increasing electron energy losses in inelastic collisions with water molecules. For constant power input, the increased electron energy losses and *T*_e_ are balanced by a decrease in *n*_e_ with increasing H_2_O content.

The effect of these changes on the total rate coefficient for dissociation *k*_diss_ = *k*_14_ + *k*_15_ + *k*_16_ and the dissociation frequency *R* = *k*_diss_*n*_e_ is shown in Fig. 11(b). Due to the variation in *T*_e_, *k*_diss_ increases with increasing humidity content, exhibiting a similar trend to the OH densities shown in Fig. 9. Here, the transition from a fast to a slow increase also occurs around 3000 ppm. The dissociation frequency *R* exhibits a peak at this humidity content, which represents the optimum between the increasing *T*_e_ and decreasing *n*_e_. Thus, the origin of the transition between fast and slow increase in OH density with increasing humidity content is a result of the transition between an increasing dissociation frequency below 3000 ppm H_2_O to a decreasing dissociation frequency above 3000 ppm H_2_O. Overall, the dissociation rate *R* × *n*_H_2_O_, and therefore the OH density, increases over the whole range due to the increasing value of *n*_H_2_O_. Based on this discussion, the differing H_2_O contents at which the transition occurs in the experiment and simulation may indicate that the rate of electron energy loss with increasing H_2_O content is misrepresented in the simulation. Another reason for the discrepancy between the simulated and measured trend in OH densities at higher water contents might be due to an additional consumption mechanism for OH, which is not taken into account in this work, such as the population of vibrationally excited states, which would also scale with *T*_e_.

### 4.3 O densities as a function of humidity content

**Fig. 12:**
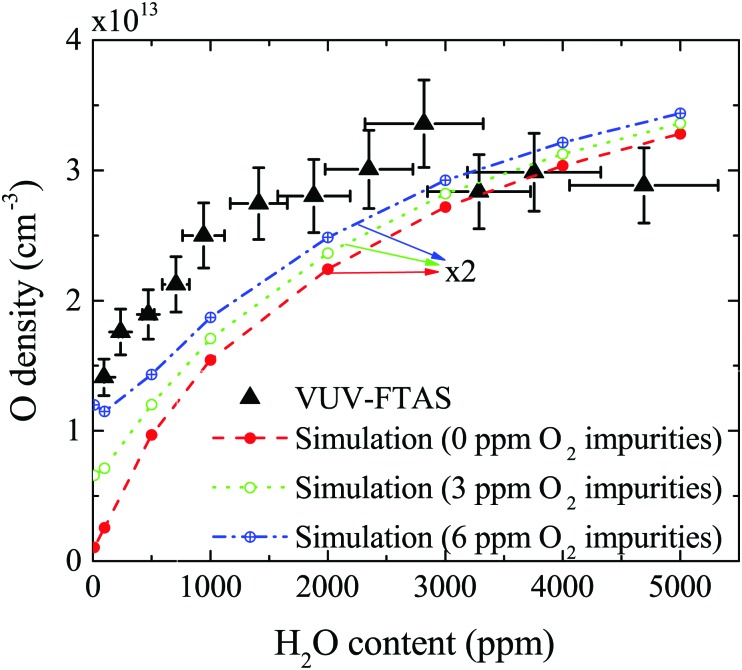
Absolute O density as a function of the water content in the He feed gas, in the centre of the plasma channel (at position *x* = 1.2 cm). Conditions are 5 slm total He flow and 10 W cm^–3^ plasma power. Simulations are also shown for different impurity level concentrations of O_2_ which may be present in the feed gas.

The absolute O density measured by VUV-FTAS in the centre of the discharge (at *x* = 1.2 cm, triangles) as a function of H_2_O content in the feed gas is shown in Fig. 12. The O density increases with increasing H_2_O content and approaches a steady-state value of around 3 × 10^13^ cm^–3^. The simulated O density also shows an increase towards higher H_2_O admixtures, however, the O densities continue to increase more significantly at higher H_2_O content than the experimental results. This is similar to the case of the OH density discussed earlier. Simulated O densities are around a factor 2 lower than those obtained experimentally. The measured densities agree well with previous measurements in a similar source using two-photon absorption laser induced fluorescence.^34^

**Fig. 13:**
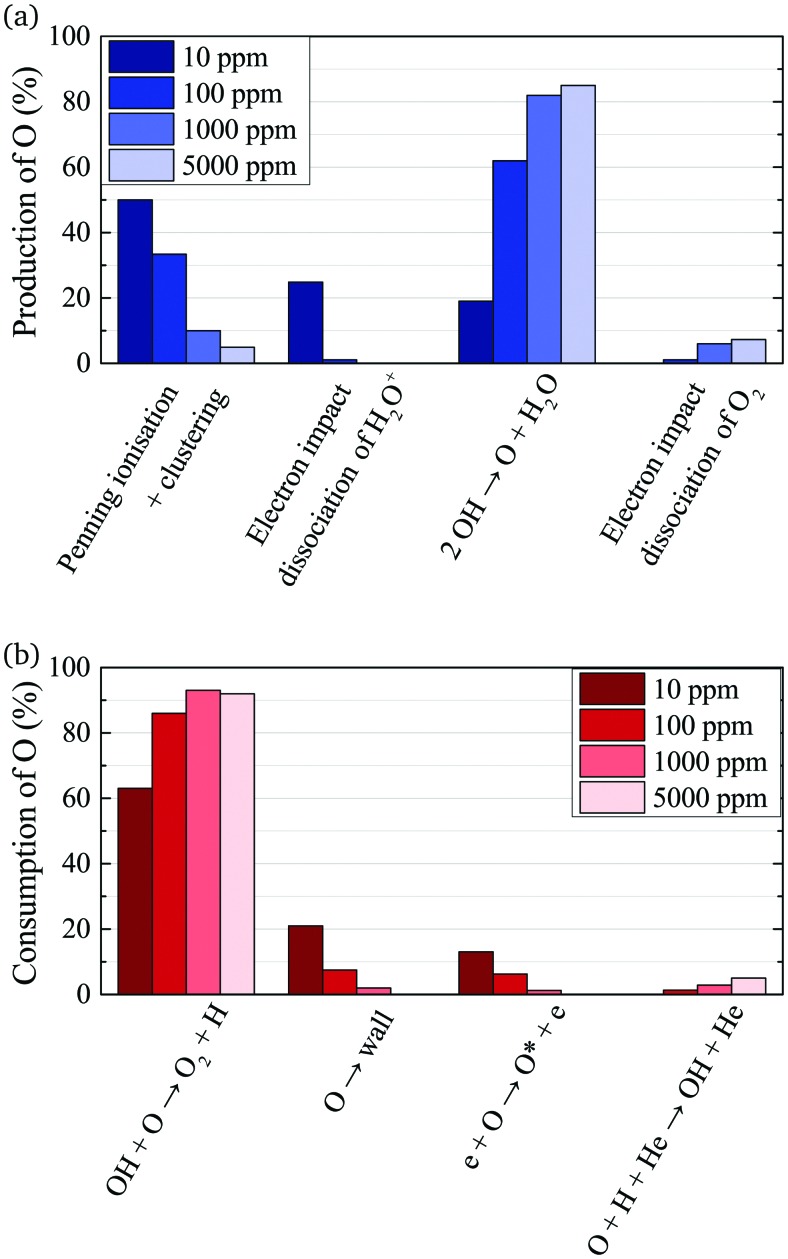
Dominant production (a) and consumption (b) pathways of O for different admixtures of H_2_O. The rates from which percentages are calculated are averaged over the whole discharge channel (0–2.4 cm, without effluent region).

A possible explanation for the difference in absolute O densities and trends between the experiment and simulation may be limitations in the global model, particularly the accuracy of the rate coefficients used, as discussed earlier. O is not directly produced from H_2_O due to electron or heavy particle impact dissociation in significant amounts at the electron temperature of interest. As a result, O must be formed in a process taking at least two steps, meaning that the uncertainties in multiple rate coefficients will play a role in determining the uncertainty in the simulated O density. As a result, the simulated O density is likely to have a larger uncertainty than the simulated OH density, whose dominant formation occurs directly from electron collisions with H_2_O. As shown in Fig. 13, the dominant production mechanism of O is *via* recombination of two OH molecules to form H_2_O and O. At lower H_2_O contents, O is also formed through processes involving positive ion water clusters:28
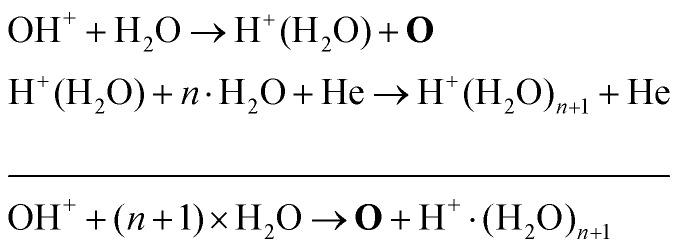



With increasing H_2_O admixture the formation of O_2_ is also increased. As a result, electron impact dissociation of O_2_ becomes a more important production pathway for O:

29 e + O_2_ → 2**O** + e (6 eV)

30 e + O_2_ → **O** + O(^1^D) + e (8.4 eV)

Where the numbers in brackets represent the electron energy thresholds for these reactions. O is mainly consumed by reactions involving OH forming O_2_ and H (eqn (27)).

The measured and simulated O densities show an increased discrepancy towards smaller H_2_O admixtures <1000 ppm. A possible explanation for this might lie in the presence of unintentional air impurities in the experiment, which have been found previously to be able to influence the chemical kinetics in atmospheric pressure plasmas.^75^–^77^ For the measurement of O, we use helium with a purity level of 99.999%, whereas the main impurities are H_2_O (3 ppm) and O_2_ (2 ppm). Additional small impurities could arise from residual gases in the feed gas line. Simulations for two different non-zero O_2_ impurity concentrations in the order of typical O_2_ impurities originating from the feed gas supply are shown in Fig. 12. Particularly at low H_2_O content, these impurities lead to an increase of O compared to the simulation without O_2_ added as an impurity. Since the density of O produced from H_2_O is low, typically a few ppm, even small O_2_ containing impurities can significantly influence the O density produced in the plasma. At high H_2_O content, the influence of O_2_ impurities on the O density is smaller, and the plasma chemistry is dominated by hydrogen containing species.

### 4.4 Numerical investigation of the production of longer-lived species

**Fig. 14:**
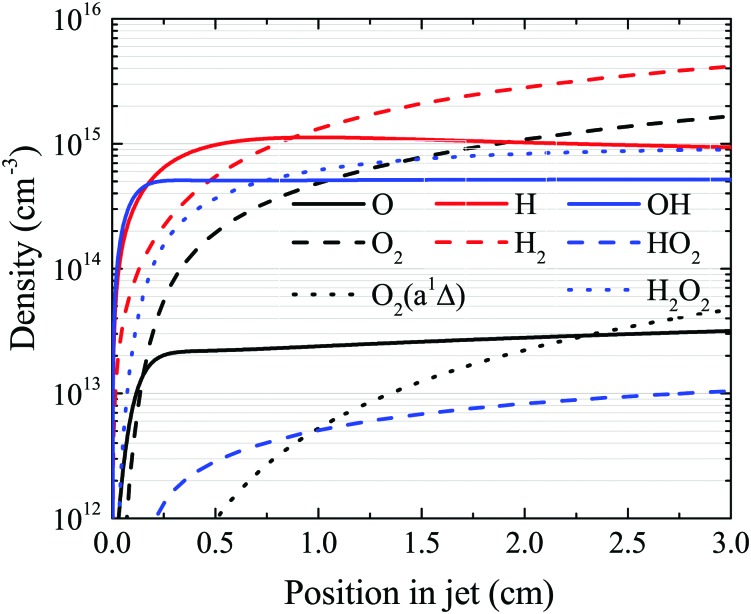
Spatial development for several species of interest as a function of position in the plasma channel, under the same conditions shown in Fig. 7 (18 W cm^–3^ plasma power, 5400 ppm humidity).

The OH density reaches a steady-state value in the simulation well before the end of the plasma channel in both simulation and experiment. Particularly at higher H_2_O content, this is a result of OH being primarily produced by direct electron impact dissociation of H_2_O in a one step process (eqn (14)) and is consumed in interactions with other OH molecules. Atomic hydrogen behaves similarly, being produced mainly by electron impact dissociation and consumed *via* interactions with surfaces,^71^ both of which occur relatively quickly. However, other species do not reach a steady state within the length of the plasma channel, and instead continuously increase in density up to the outlet of the plasma source. This is particularly true for slowly forming, long-lived species such as O_2_, H_2_O_2_ and H_2_, as shown in Fig. 14. This finding suggests that the length of the plasma source, or the gas flow rate, and therefore the residence time of the gas, can be used to control the ratio of different species densities by taking advantage of the different timescales required for them to reach steady-state.

First, we will discuss the formation of O in more detail. The density of O does not reach a steady-state value in the simulation within the plasma channel for most investigated conditions using a He–H_2_O gas mixture. Long timescales for simulations of atmospheric pressure He–H_2_O plasmas to reach steady-state have also been found by others.^78^ This is in contrast to the case where similar sources are operated in He–O_2_ mixtures.^79^ In the work described in ref. 79, O densities approach steady-state towards the end of the plasma channel of the AAPPJ. In Fig. 14, O densities are increasing sharply within the first few millimetres of the channel, and then at a lower rate up to the end of the channel. Therefore, O densities follow a similar dependence as the OH densities also shown in Fig. 14. This is not surprising when considering that both the dominant production and consumption pathways are related to OH, *i.e.* production by reactions of two OH molecules to form H_2_O and O (eqn (19)), and consumption *via* collisions with OH to form O_2_ and H (eqn (27)). The fact that O is still building up within the channel, while OH approaches a steady-state value, is due to the continuous build-up of O_2_ in the channel, also shown in Fig. 14. Electron impact dissociation of O_2_ (eqn (29) and (30)) provides an additional formation mechanism for O further into the channel, although eqn (19) and (27) are still the dominant production and consumption pathways for O. Overall, this leads to a slow increase of the O density while the O_2_ density continues to increase.

The formation of species that reach steady-state on timescales longer than the residence time in the discharge channel are usually comprised of a complex multi-step processes. As an example, we demonstrate the dominant pathways for formation of O_2_, which is an important precursor for the formation of excited states of O_2_, such as O_2_(a ^1^Δ). Since O_2_ is a slowly forming species, we look at dominant production and consumption pathways for a longer timescale *τ*_p_ than those previously given in Table 2. The time scale of interest in the simulation is chosen so that only He, H_2_O, O_2_, O_2_(a ^1^Δ), H_2_, and H_2_O_2_ are treated as long-lived species, in accordance with previous studies.^52^ The computational lifetimes of the shortest-lived species of these six are listed in Table 3 for different H_2_O contents.

**Table 3:** Lifetimes of the shortest-lived species out of He, H_2_O, O_2_, O_2_(a ^1^Δ), H_2_, and H_2_O_2_ for different humidity contents in the plasma

H_2_O content (ppm)	O_2_(a ^1^Δ) lifetime (ms)	H_2_O_2_ lifetime (ms)
100	2.78	4.38
1000	2.74	2.74
10 000	4.55	1.35

The two main net production reactions for the formation of molecular oxygen are found to be

31 2H_2_O → 2H_2_ + **O**_2_

32 H_2_O_2_ → H_2_ + **O**_2_

Many different pathways are possible in order to obtain these net reactions. A few examples of these pathways are33
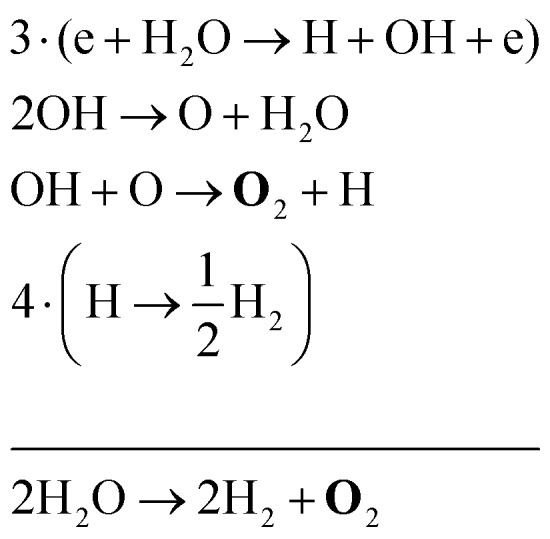

34
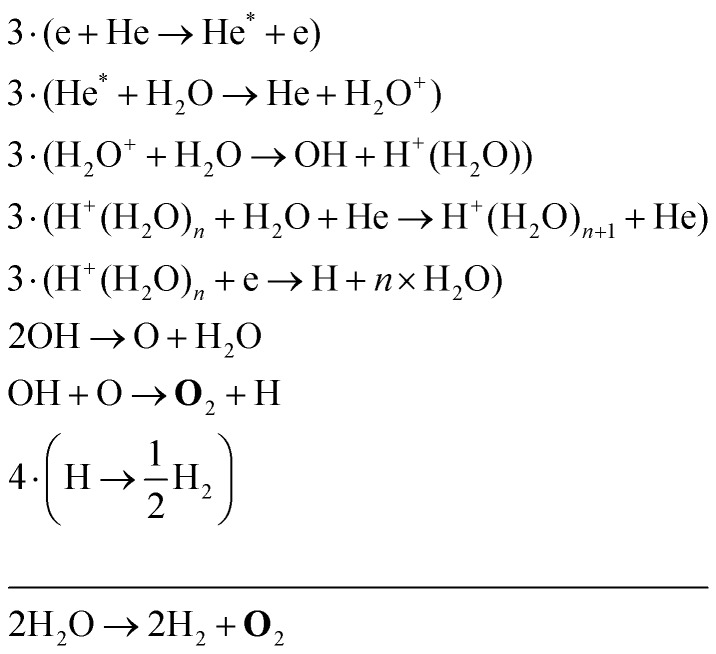

35
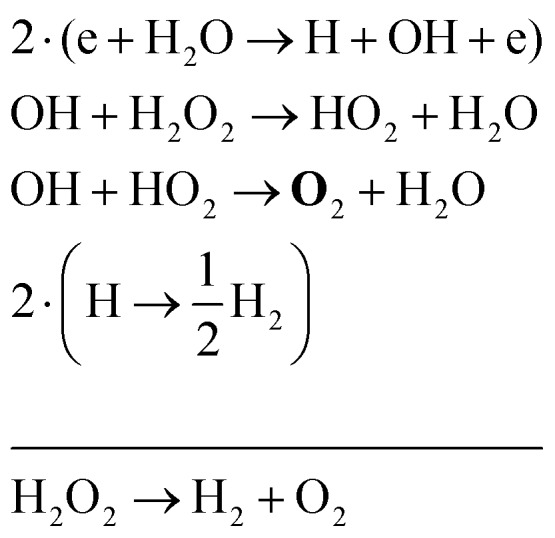



Note that eqn (33) and (34) have the same net production reaction, although the intermediate steps towards the formation of O_2_ (*i.e.* the pathway) are different. Eqn (33) starts with the electron impact dissociation of H_2_O molecules. The reaction of OH with OH and O leads to the formation of O_2_. The H atoms formed recombine at surfaces to form H_2_.

Eqn (34) is the same net reaction, however, the step-by-step analysis reveals a different pathway. In this case, H_2_O molecules are first ionised *via* Penning ionisation with He*. H_2_O^+^ ions then start accumulating more H_2_O molecules in a clustering process, where OH is produced. Similar to the previous pathways, the reaction of OH with OH and O lead to the formation of O_2_. The cluster ions produced are consumed by dissociative recombination with electrons, and H, which is formed in that process, is lost by surface recombination. Other pathways exists that involve the formation of clusters, but are not explicitly discussed here.

Eqn (35) has a different net production reaction than the others. In this case, OH produced from electron impact dissociation reacts with H_2_O_2_ to form reactive HO_2_, which then forms O_2_ during reactions with OH.

## 5 Conclusions

In this work, the chemical kinetics in an rf atmospheric pressure plasma with humidity are investigated using experimental and numerical techniques. By using this combination, computed species densities are benchmarked against experimental densities. The simulations are then used to reveal the dominant formation pathways of species of interest, here OH and O, and longer lived species such as O_2_, which is an important precursor for the formation of its excited states, such as O_2_(a ^1^Δ). This work provides a detailed understanding of chemical kinetics in the active plasma. In many applications, reactive species will exit the plasma source and transit into an effluent region where they will mix with ambient air, where their chemical kinetics will differ. While this is not considered in this work, the results presented here provide a basis to be built on in future work to understand reactive species kinetics in this transition region.

Absolute number densities of O and OH are determined experimentally using VUV high-resolution Fourier-transform absorption spectroscopy, UV broad-band absorption spectroscopy, and numerically by using the 0-D plasma chemical kinetics code GlobalKin.

Absolute OH densities and formation pathways are investigated as a function of position in the discharge. Three different regions can be identified *i.e.* (a) a strong increase of OH density in the first few millimeters of the plasma channel, (b) a quasi steady-state region, and (c) a rapid drop of OH density in the plasma effluent region. During the fast increase and steady-state regions, OH is mainly produced *via* fast processes such as electron impact dissociation of H_2_O, and consumed predominantly *via* reactions with other OH molecules to form H_2_O_2_ or H_2_O. These relatively simple chemical kinetics make it possible for OH to reach an equilibrium value within the plasma channel.

Other species, whose densities have not been measured, are investigated numerically as a function of position in the plasma channel. Simulation results show that the H density approaches a steady-state value within the plasma channel, similarly to OH as discussed previously, as it is mostly formed directly *via* electron impact dissociation of water, and consumed at surfaces to form stable H_2_. However, most other species generated in the He–H_2_O plasma studied in this work do not reach a steady-state value within the length of the plasma channel due to more complex formation mechanisms. This has been shown using O_2_ as an example. Therefore, the length of the plasma source could be used as a control parameter to tune the chemical composition of the gas at the end of the plasma jet for applications.

Both OH and O densities are also investigated as a function of the humidity content in the He feed gas. It is found, both in experiments and simulations, that O and OH densities increase non-linearly with increasing feed gas humidity, offering the possibility of tailoring reactive species densities by changing the feed gas composition.

The maximum OH density is on the order of 3–4 × 10^14^ cm^–3^ (13–17 ppm). It is found that at very low water content, OH is mainly produced *via* reactions between H_2_O^+^ and water molecules to form OH and protonated water clusters of the form H^+^·(H_2_O)_*n*_, while electron impact dissociation of H_2_O becomes an increasingly important production pathway with increasing water content. The main loss channel for OH at all H_2_O contents is recombination to form H_2_O_2_.

The maximum O density on the other hand is found to be in the order of 3 × 10^13^ cm^–3^ (1.3 ppm). Recombination of two OH molecules is the most important production process for O at all H_2_O contents, while at very low water content, OH is also strongly produced *via* reactions between OH^+^ and water molecules to form O and protonated water clusters. Since the dominant destruction pathway of O is recombination with OH to form O_2_ and H, the formation of O is strongly coupled to the OH density in the gas flow. At higher H_2_O concentrations, electron impact dissociation of accumulated O_2_ can also contribute to the production of O. It is also found that towards low H_2_O content, production of O from air impurities in the ppm range originating from the feed gas can increase the O density *via* direct electron impact dissociation of O_2_. Towards higher H_2_O admixtures, this effect becomes less significant due to increased production *via* collisions involving OH. Therefore, larger amounts of purposely admixed molecules lead to a better control of the plasma properties and reactive species than operating the source with small or no intentional admixtures.

## Conflicts of interest

There are no conflicts to declare.

## Appendices

### Appendix

#### Appendix

##### A. Reaction mechanism

Table 4 shows wall recombination coefficients and return species for the simulations used in this work.

**Table 4:** Wall sticking coefficients and return fractions for various species considered in this work

Species	*γ*	Return species	Ref.
He_2_*	1.00	2He	est.
O	0.02	0.5O_2_	48 and 80
O_2_(a ^1^Δ)	0.0004	O_2_	48
O_2_(b ^1^Σ)	0.02	O_2_	48 and 80
H	0.03	0.5H_2_	48 and 81
N	1.00	0.5N_2_	est.
He_2_^+^	1.00	2He	est.
O_4_^+^	1.00	2O_2_	est.
N_4_^+^	1.00	2N_2_	est.
O_2_^+^(H_2_O)	1.00	O_2_ + H_2_O	est.
H_2_O^+^(H_2_O)	1.00	2H_2_O	est.

Table 5 shows electron impact reactions used in this work. Reaction rate coefficients are either taken from the literature, or calculated by the GlobalKin two-term Boltzmann equation solver. For the latter, reaction rate coefficients are indicated as *f*(*E*), and collisional cross sections are taken from the indicated literature. Electron impact cross-sections are taken from several databases, for example He,^82^,^83^ or H_2_O.^84^ Although not all of the reactions from these databases are included in the plasma-chemical reaction mechanism shown here, they are still accounted for in the Boltzmann solver calculation for the electron energy distribution function and electron transport coefficients. Any other approach to obtain reaction rate coefficients is denoted by footnotes.

**Table 5:** Electron collisions

No.	*E*_Thr_ (eV)	Reaction (rxn)	Rate^*a*^ ^,^^*b*^	Ref.
Elastic scattering and momentum transfer				
1	0.00	e + He → He + e	*f*(*E*)	82 and 83
2	0.00	e + H_2_O → H_2_O + e	*f*(*E*)	84 and 89
3	0.00	e + O_2_ → O_2_ + e	*f*(*E*)	90

Electron impact excitation and ionisation				
4	19.80	e + He → He* + e	*f*(*E*)	82 and 83
5	20.62	e + He → He* + e	*f*(*E*)	82 and 83
6	24.58	e + He → He^+^ + e	*f*(*E*)	82 and 83
7	4.77	e + He* → He^+^ + 2e	*f*(*E*)	91 ^*c*^
8	3.90	e + He_2_* → He_2_^+^ + 2e	2.06 × 10^–13^e^–4.28/*T*_e_^	92 ^*d*^
9	0.20	e + H_2_O → H_2_O + e	*f*(*E*)	84 ^*e*^
10	0.45	e + H_2_O → H_2_O + e	*f*(*E*)	84 ^*e*^
11	0.47	e + H_2_O → H_2_O + e	*f*(*E*)	84 ^*e*^
12	13.50	e + H_2_O → H_2_O^+^ + 2e	*f*(*E*)	84
13	13.50	e + OH → OH^+^ + 2e	*f*(*E*)	93
14	1.97	e + O → O(^1^D) + e	*f*(*E*)	94
15	4.19	e + O → O(^1^S) + e	*f*(*E*)	94
16	13.62	e + O → O^+^ + 2e	*f*(*E*)	94
17	11.65	e + O(^1^D) → O^+^ + 2e	*f*(*E*)	91 ^*c*^
18	9.43	e + O(^1^S) → O^+^ + 2e	*f*(*E*)	95 ^*c*^
19	0.02	e + O_2_ → O_2_ + e	*f*(*E*)	90 ^*f*^
20	0.19	e + O_2_ → O_2_ + e	*f*(*E*)	90 ^*e*^
21	0.19	e + O_2_ → O_2_ + e	*f*(*E*)	90 ^*e*^
22	0.38	e + O_2_ → O_2_ + e	*f*(*E*)	90 ^*e*^
23	0.38	e + O_2_ → O_2_ + e	*f*(*E*)	90 ^*e*^
24	0.57	e + O_2_ → O_2_ + e	*f*(*E*)	90 ^*e*^
25	0.75	e + O_2_ → O_2_ + e	*f*(*E*)	90 ^*e*^
26	0.98	e + O_2_ → O_2_(a ^1^Δ) + e	*f*(*E*)	90
27	1.63	e + O_2_ → O_2_(b ^1^Σ) + e	*f*(*E*)	90
28	4.50	e + O_2_ → O_2_ + e	*f*(*E*)	90 ^*g*^
29	12.06	e + O_2_ → O_2_^+^ + e	*f*(*E*)	90
30	0.02	e + O_2_(a ^1^Δ) → O_2_(a ^1^Δ) + e	*f*(*E*)	As rxn 19^*h*^
31	0.19	e + O_2_(a ^1^Δ) → O_2_(a ^1^Δ) + e	*f*(*E*)	As rxn 20^*h*^
32	0.19	e + O_2_(a ^1^Δ) → O_2_(a ^1^Δ) + e	*f*(*E*)	As rxn 21^*h*^
33	0.38	e + O_2_(a ^1^Δ) → O_2_(a ^1^Δ) + e	*f*(*E*)	As rxn 22^*h*^
34	0.38	e + O_2_(a ^1^Δ) → O_2_(a ^1^Δ) + e	*f*(*E*)	As rxn 23^*h*^
35	0.57	e + O_2_(a ^1^Δ) → O_2_(a ^1^Δ) + e	*f*(*E*)	As rxn 24^*h*^
36	0.75	e + O_2_(a ^1^Δ) → O_2_(a ^1^Δ) + e	*f*(*E*)	As rxn 25^*h*^
37	0.65	e + O_2_(a ^1^Δ) → O_2_(b ^1^Σ) + e	*f*(*E*)	96 ^*i*^
38	3.52	e + O_2_(a ^1^Δ) → O_2_ + 2e	*f*(*E*)	As rxn 28^*h*^
39	11.08	e + O_2_(a ^1^Δ) → O_2_^+^ + e	*f*(*E*)	As rxn 29^*h*^
40	0.02	e + O_2_(b ^1^Σ) → O_2_(b ^1^Σ) + e	*f*(*E*)	As rxn 19^*h*^
41	0.19	e + O_2_(b ^1^Σ) → O_2_(b ^1^Σ) + e	*f*(*E*)	As rxn 20^*h*^
42	0.19	e + O_2_(b ^1^Σ) → O_2_(b ^1^Σ) + e	*f*(*E*)	As rxn 21^*h*^
43	0.38	e + O_2_(b ^1^Σ) → O_2_(b ^1^Σ) + e	*f*(*E*)	As rxn 22^*h*^
44	0.38	e + O_2_(b ^1^Σ) → O_2_(b ^1^Σ) + e	*f*(*E*)	As rxn 23^*h*^
45	0.57	e + O_2_(b ^1^Σ) → O_2_(b ^1^Σ) + e	*f*(*E*)	As rxn 24^*h*^
46	0.75	e + O_2_(b ^1^Σ) → O_2_(b ^1^Σ) + e	*f*(*E*)	As rxn 25^*h*^
47	2.87	e + O_2_(b ^1^Σ) → O_2_ + e	*f*(*E*)	As rxn 28^*h*^
48	10.43	e + O_2_(b ^1^Σ) → O_2_^+^ + 2e	*f*(*E*)	As rxn 29^*h*^

Super-elastic collisions				
49	–19.80	e + He* → He + e	*f*(*E*)	82 and 83 ^*j*^
50	–1.97	e + O(^1^D) → O + e	*f*(*E*)	94 ^*j*^
51	–4.19	e + O(^1^S) → O + e	*f*(*E*)	94 ^*j*^
52	–0.98	e + O_2_(a ^1^Δ) → O_2_ + e	*f*(*E*)	90 ^*j*^
53	–1.63	e + O_2_(b ^1^Σ) → O_2_ + e	*f*(*E*)	90 ^*j*^
54	–0.65	e + O_2_(b ^1^Σ) → O_2_(a ^1^Δ) + e	*f*(*E*)	As rxn 37^*j*^

Electron impact dissociation				
55	0.00	e + He_2_* → 2He + e	3.8 × 10^–15^	97
56	13.50	e + H_2_O → O(^1^S) + 2H + e	*f*(*E*)	84 and 98
57	7.62	e + H_2_O → H + OH + e	*f*(*E*)	84 and 99
58	9.00	e + H_2_O → H + OH + e	*f*(*E*)	84
59	13.00	e + H_2_O → H_2_ + O(^1^D) + e	2.42 × 10^–14^*T*_e_^–0.062^e^–22.42/*T*_e_^	78 ^*k*^
60	8.80	e + H_2_ → 2H + e	*f*(*E*)	100
61	11.37	e + H_2_ → 2H + e	*f*(*E*)	101
62	12.96	e + OH → O + H + e	*f*(*E*)	102 ^*l*^
63		e + H_2_O_2_ → 2OH + e	2.36 × 10^–15^	103 ^*m*^
64	6.00	e + O_2_ → O + O + e	*f*(*E*)	90
65	8.40	e + O_2_ → O(^1^D) + O + e	*f*(*E*)	90
66	10.00	e + O_2_ → O(^1^D) + O + e	*f*(*E*)	90
67	5.02	e + O_2_(a ^1^Δ) → O + O + e	*f*(*E*)	As rxn 64^*h*^
68	7.42	e + O_2_(a ^1^Δ) → O(^1^D) + O + e	*f*(*E*)	As rxn 65^*h*^
69	9.02	e + O_2_(a ^1^Δ) → O(^1^D) + O + e	*f*(*E*)	As rxn 66^*h*^
70	4.37	e + O_2_(b ^1^Σ) → O + O + e	*f*(*E*)	As rxn 64^*h*^
71	6.77	e + O_2_(b ^1^Σ) → O(^1^D) + O + e	*f*(*E*)	As rxn 65^*h*^
72	8.37	e + O_2_(b ^1^Σ) → O(^1^D) + O + e	*f*(*E*)	As rxn 66^*h*^
73	2.60	e + O_3_ → O + O_2_ + e	1.7 × 10^–14^*T*_e_^–0.57^e^–2.48/*T*_e_^	74 and 104
74	5.72	e + O_3_ → O(^1^D) + O_2_(a ^1^Δ) + e	3.22 × 10^–13^*T*_e_^–1.18^e^–9.17/*T*_e_^	74 and 104

Dissociative ionisation				
75	17.50	e + H_2_O → OH^+^ + H + 2e	*f*(*E*)	84
76	25.00	e + H_2_O → O^+^ + 2H + 2e	*f*(*E*)	84

(Dissociative) electron attachment				
77	5.30	e + H_2_O → OH + H^–^	*f*(*E*)	84 and 105
78	4.43	e + H_2_O → H_2_ + O^–^	*f*(*E*)	84 and 105
79	4.30	e + H_2_O → H + OH^–^	*f*(*E*)	84 and 105
80	0.00	e + H_2_O_2_ → H_2_O + O^–^	*f*(*E*)	106
81	0.00	e + H_2_O_2_ → OH + OH^–^	*f*(*E*)	106
82	5.38	e + O_2_ → O + O^–^	*f*(*E*)	90
83	3.50	e + O_2_(a ^1^Δ) → O + O^–^	*f*(*E*)	107
84	2.85	e + O_2_(b ^1^Σ) → O + O^–^	*f*(*E*)	As rxn 83^*h*^
85	0.00	e + O_3_ → O_2_ + O^–^	*f*(*E*)	108
86	0.25	e + O_3_ → O_2_^–^ + O	*f*(*E*)	108

Electron detachment				
87	1.55	e + H^–^ → H + e + e	*f*(*E*)	109
88	3.37	e + OH^–^ → OH + e + e	*f*(*E*)	110
89	2.70	e + O^–^ → O + e + e	*f*(*E*)	111
90	4.00	e + O_2_^–^ → O_2_ + e + e	*f*(*E*)	174

Recombination				
91	0.00	e + H_2_O^+^ → H + OH	8.6 × 10^–14^*T*_e_^–0.5^	112 and 113
92	0.00	e + H_2_O^+^ → 2H + O	3.05 × 10^–13^*T*_e_^–0.5^	112 and 113
93	0.00	e + H_2_O^+^ → H_2_ + O	3.87 × 10^–14^*T*_e_^–0.5^	112 and 113
94	0.00	e + H^+^·(H_2_O) → H + H_2_O	7.09 × 10^–14^*T*_e_^–0.5^	112, 114 and 115
95	0.00	e + H^+^·(H_2_O) → OH + H_2_	5.37 × 10^–14^*T*_e_^–0.5^	112, 114 and 115
96	0.00	e + H^+^·(H_2_O) → OH + 2H	3.05 × 10^–13^*T*_e_^–0.5^	112, 114 and 115
97	0.00	e + H^+^·(H_2_O)_2_ → H + 2H_2_O	1.84 × 10^–12^*T*_e_^–0.08^	116
98	0.00	e + H^+^·(H_2_O)_3_ → 3H_2_O + H	2.24 × 10^–12^*T*_e_^–0.08^	116
99	0.00	e + H^+^·(H_2_O)_4_ → 4H_2_O + H	3.6 × 10^–12^	116
100	0.00	e + H^+^·(H_2_O)_5_ → 5H_2_O + H	4.1 × 10^–12^	117
101	0.00	e + H^+^·(H_2_O)_6_ → 6H_2_O + H	5.13 × 10^–12^	117
102	0.00	e + H^+^·(H_2_O)_7_ → 7H_2_O + H	1.0 × 10^–12^	117
103	0.00	e + H^+^·(H_2_O)_8_ → 8H_2_O + H	4.1 × 10^–12^	As rxn 100
104	0.00	e + H^+^·(H_2_O)_9_ → 9H_2_O + H	4.1 × 10^–12^	As rxn 100
105	0.00	e + H_2_O^+^·(H_2_O) → H + OH + H_2_O	9.63 × 10^–13^*T*_e_^–0.2^	118 ^*n*^
106	0.00	e + O_2_^+^ → 2O	3.72 × 10^–15^*T*_e_^–0.7^	119
107	0.00	e + O_2_^+^ → O + O(^1^D)	7.44 × 10^–15^*T*_e_^–0.7^	119 and 120
108	0.00	e + O_2_^+^ → 2O(^1^D)	7.44 × 10^–15^*T*_e_^–0.7^	119 and 120
109	0.00	e + O_2_^+^·(H_2_O) → O_2_ + H_2_O	7.22 × 10^–13^*T*_e_^–0.2^	118
110	0.00	e + O_4_^+^ → 2O + O_2_	5.17 × 10^–14^*T*_e_^–1.0^	118
111	0.00	e + O_4_^+^ → 2O_2_	2.76 × 10^–13^*T*_e_^–0.5^	86

^*a*^In m^3^ s^–1^ and m^6^ s^–1^ for two- and three-body processes, respectively.

^*b*^ *f*(*E*) denotes rate coefficients are calculated by the internal GlobalKin two-term Boltzmann equation solver using cross sections obtained from the indicated literature.

^*c*^Cross sections are calculated from an expression in cited reference.

^*d*^Calculated assuming a Maxwell distribution function and cross sections from the given reference.

^*e*^Vibrational excitation cross section included in cross section set for two-term Boltzmann solver. Vibrational states not simulated self-consistently in reaction kinetics.

^*f*^Rotational excitation cross section included in cross section set for two-term Boltzmann solver. Rotations states not simulated self-consistently in reaction kinetics.

^*g*^Electronic excitation cross section included in cross section set for two-term Boltzmann solver. This electronic state is not simulated self-consistently in reaction kinetics.

^*h*^Cross section estimated by shifting and scaling the corresponding cross section for the ground state by the excitation threshold of the excited state.

^*i*^Born–Bethe fit to data in the cited reference.

^*j*^Obtained from reverse process by detailed balance.

^*k*^In the reference reaction rates were calculated using Bolsig+^121^ and cross sections obtained from the Morgan database^122^ for a He/H_2_O plasma.

^*l*^Cross section assumed to be the same as that for CO.

^*m*^Value is approximated in reference based on cross section for electron impact dissociation of O_2_.

^*n*^Value is estimated in reference.

Table 6 shows reaction rate coefficients for ion–ion recombination processes. It is generally known that ion–ion recombination processes can occur both as two- or three-body processes, depending on the gas pressure. Two-body reaction rate coefficients for several different gases have been obtained in ref. 85 at low pressure, and found to be in the order of 10^–13^ m^3^ s^–1^ or lower. Taking into account the He density at atmospheric pressure and at 315 K, and the rate coefficient for three-body ion–ion recombination proposed by Kossyi,^86^ the effective two-body reaction rate amounts to a value in the order of 10^–12^ m^3^ s^–1^. Due to this higher effective rate coefficient under our conditions, we only include three-body ion–ion recombination rate coefficients in this work. These reactions are found to be particularly important for the destruction of the higher-mass water clusters, which are abundant at higher H_2_O admixtures. Similar observations have been made by Liu *et al.*,^48^ who, after an analysis of the robustness of their chemistry model, only included a few ion–ion recombination reactions in their simplified models, a large fraction of which were three-body recombination processes for the collisions of higher mass cluster ions. We also found that under our conditions, ion–ion recombination between positive He ions and negative ions are negligible due to the rapidly decreasing He ion density with increasing water content, which again is in accordance with the findings of Liu *et al.*,^48^ and the fact that He ions undergo charge exchange reactions with most neutral species due to their high ionisation potential.

**Table 6:** Ion–ion chemistry

No.	Reaction (rxn)	Rate^*a*^	Ref.
Three-body collisions			
112–131	A^+^ + B^–^ + He → A + B + He	2.0 × 10^–37^*T*_0_^–2.5^	86 ^*b*^ ^,^ ^*c*^
132–136	A_4_^+^ + B^–^ + He → 2A_2_ + B + He	2.0 × 10^–37^*T*_0_^–2.5^	86 ^*b*^ ^,^ ^*d*^
137–148	A^+^ + OH^–^·(H_2_O)_*n*_ + He → A + OH + *n* × H_2_O + He	2.0 × 10^–37^*T*_0_^–2.5^	86 ^*b*^ ^,^ ^*e*^
149–151	A_4_^+^ + OH^–^·(H_2_O)_*n*_ + He → 2A_2_ + OH + *n* × H_2_O + He	2.0 × 10^–37^*T*_0_^–2.5^	86 ^*b*^ ^,^ ^*f*^
152–187	H^+^·(H_2_O)_*m*_ + B^–^ + He → *m* × H_2_O + H·B	2.0 × 10^–37^*T*_0_^–2.5^	86 ^*b*^ ^,^ ^*g*^
188–214	H^+^·(H_2_O)_*m*_ + OH^–^·(H_2_O)_*n*_ + He → (*m* + *n* + 1) × H_2_O + He	2.0 × 10^–37^*T*_0_^–2.5^	86 ^*b*^ ^,^ ^*h*^
215–223	H^+^·(H_2_O)_*m*_ + H_2_O_2_^–^ + He → (*m* + 1) × H_2_O + OH	2.0 × 10^–37^*T*_0_^–2.5^	86 ^*b*^ ^,^ ^*i*^
224–228	H_2_O^+^·H_2_O + B^–^ + He → 2H_2_O + B + He	2.0 × 10^–37^*T*_0_^–2.5^	86 ^*b*^ ^,^ ^*j*^
229–231	H_2_O^+^·H_2_O + OH^–^·(H_2_O)_*n*_ + He → (*n* + 2) × H_2_O + OH + He	2.0 × 10^–37^*T*_0_^–2.5^	86 ^*b*^ ^,^ ^*k*^
232–236	O_2_^+^·(H_2_O) + B^–^ + He → H_2_O + O_2_ + B	2.0 × 10^–37^*T*_0_^–2.5^	86 ^*b*^ ^,^ ^*j*^
237–239	O_2_^+^·(H_2_O) + OH^–^·(H_2_O)_*n*_ + He → (*n* + 1) × H_2_O + O_2_ + OH	2.0 × 10^–37^*T*_0_^–2.5^	86 ^*b*^ ^,^ ^*k*^

^*a*^In m^6^ s^–1^.

^*b*^Value estimated in reference.

^*c*^For A = O, O_2_, OH, H_2_O and B = O, O_2_, H, OH, H_2_O_2_.

^*d*^For A = O and B = O, O_2_, H, OH, H_2_O_2_.

^*e*^For A = O, O_2_, OH, H_2_O and *n* = 1–3.

^*f*^For A = O and *n* = 1–3.

^*g*^For *m* = 1–9 and B = O, H, OH.

^*h*^For *m* = 1–9 and *n* = 1–3.

^*i*^For *m* = 1–9.

^*j*^For B = O, O_2_, H, OH, H_2_O_2_.

^*k*^For *n* = 1–3.

Table 7 shows reaction rate coefficients for collisions between ions and neutrals. In this table a number of three-body processes are included. Three-body processes are typically characterised by a pressure dependence. The nature of these reactions mean that this pressure dependence normally takes the form of a curve exhibiting low- and high-pressure limits. In the low-pressure limit the effective rate coefficient (*i.e.* the three-body rate coefficient multiplied by the third body density) is linear with the third body density. In the high pressure limit the effective rate coefficient is independent of the density of the third body. In the region between the two limits the effective rate coefficient is non-linear with the third body density. For a number of reactions, this transition region occurs around atmospheric pressure, therefore effective rate coefficients must be calculated using available knowledge of the high and low pressure limits. The coefficients which have been explicitly calculated for atmospheric pressure are marked as “*effective*” in Tables 7 and 8. Among these reactions is the formation and destruction of protonated water clusters H^+^·(H_2_O)_*n*_, where the rate coefficients are given by Sieck *et al.*^87^ Here, the expressions given in ref. 87 are used to calculate the effective rate coefficients for these reactions under our plasma operating conditions (atmospheric pressure, *T*_g_ = 280–350 K). Results are fitted with an Arrhenius expression, where possible, in order to keep the temperature dependence for these reactions, since the formation of cluster ions is highly temperature dependent.^87^ The rate coefficients for the formation of the two highest order clusters taken into account in this work are estimated by extrapolating the coefficients *k*3000 and *A* given by Sieck *et al.* using an exponential fit, and using constant values *n* = 16, *B* = 5000, and *k*_L_ = 10^–24^ (see Sieck *et al.*^87^ for further description of these coefficients).

**Table 7:** Ion-neutral chemistry

No.	Reaction (rxn)	Rate^*a*^	Ref.
Two-body collisions – positive ions			
240	He^+^ + OH → O^+^ + H + He	1.1 × 10^–15^*T*_0_^–0.5^	112 and 123
241	He^+^ + H_2_O → H_2_O^+^ + He	6.05 × 10^–17^*T*_0_^–0.5^	112 and 124
242	He^+^ + H_2_O → OH^+^ + H + He	2.86 × 10^–16^*T*_0_^–0.5^	112 and 124
243	He^+^ + O → O^+^ + He	5.00 × 10^–17^	125
244	He^+^ + O_2_ → O^+^ + O + He	1.1 × 10^–15^	112 and 126
245	He^+^ + O_2_ → O_2_^+^ + He	3.3 × 10^–17^	112 and 126
246	He_2_^+^ + OH → O^+^ + H + 2He	1.1 × 10^–15^	As rxn 240
247	He_2_^+^ + H_2_O → H_2_O^+^ + 2He	6.05 × 10^–17^*T*_0_^–0.5^	As rxn 241
248	He_2_^+^ + H_2_O → H^+^·(H_2_O) + 2He	2.86 × 10^–16^*T*_0_^–0.5^	As rxn 242
249	He_2_^+^ + O_2_ → O^+^ + O + 2He	1.1 × 10^–15^	As rxn 244
250	He_2_^+^ + O_2_ → O_2_^+^ + 2He	3.3 × 10^–17^	As rxn 245
251	H^+^·(H_2_O)_2_ (+ He) → H^+^·(H_2_O) + H_2_O (+ He)	Effective	87 ^*c*^ ^,^ ^*d*^
252	H^+^·(H_2_O)_3_ (+ He) → H^+^·(H_2_O)_2_ + H_2_O (+ He)	Effective	87 ^*c*^ ^,^ ^*d*^
253	H^+^·(H_2_O)_4_ (+ He) → H^+^·(H_2_O)_3_ + H_2_O (+ He)	Effective	87 ^*c*^ ^,^ ^*d*^
254	H^+^·(H_2_O)_5_ (+ He) → H^+^·(H_2_O)_4_ + H_2_O (+ He)	Effective	87 ^*c*^ ^,^ ^*d*^
255	H^+^·(H_2_O)_6_ (+ He) → H^+^·(H_2_O)_5_ + H_2_O (+ He)	Effective	87 ^*c*^ ^,^ ^*d*^
256	H^+^·(H_2_O)_7_ (+ He) → H^+^·(H_2_O)_6_ + H_2_O (+ He)	Effective	87 ^*c*^ ^,^ ^*d*^
257	H^+^·(H_2_O)_8_ (+ He) → H^+^·(H_2_O)_7_ + H_2_O (+ He)	Effective	est.^*e*^
258	H^+^·(H_2_O)_9_ (+ He) → H^+^·(H_2_O)_8_ + H_2_O (+ He)	Effective	est.^*e*^
259	OH^+^ + H_2_ → H_2_O^+^ + H	1.01 × 10^–15^	112 and 127
260	OH^+^ + OH → H_2_O^+^ + O	7.0 × 10^–16^*T*_0_^–0.5^	112 and 123
261	OH^+^ + H_2_O → H_2_O^+^ + OH	1.56 × 10^–15^	128
262	OH^+^ + H_2_O → H^+^·(H_2_O) + O	1.27 × 10^–15^	128
263	OH^+^ + O → O_2_^+^ + H	7.1 × 10^–16^	112 and 123
264	OH^+^ + O_2_ → O_2_^+^ + OH	5.9 × 10^–16^	112 and 127
265	H_2_O^+^ + H_2_ → H^+^·(H_2_O) + H	6.4 × 10^–16^	112 and 129
266	H_2_O^+^ + OH → H^+^·(H_2_O) + O	6.9 × 10^–16^*T*_0_^–0.5^	112 and 123
267	H_2_O^+^ + H_2_O → H^+^·(H_2_O) + OH	2.05 × 10^–15^	128
268	H_2_O^+^ + O → O_2_^+^ + H_2_	4.0 × 10^–17^	112 and 130
269	H_2_O^+^ + O_2_ → O_2_^+^ + H_2_O	3.3 × 10^–16^	131
270	H_2_O^+^·H_2_O + H_2_O → H^+^·(H_2_O)_2_ + OH	1.4 × 10^–15^	118
271	O^+^ + H_2_ → OH^+^ + H	1.7 × 10^–15^	112 and 132
272	O^+^ + OH → OH^+^ + O	3.6 × 10^–15^*T*_0_^–0.5^	112 and 123
273	O^+^ + OH → O_2_^+^ + H	3.6 × 10^–15^*T*_0_^–0.5^	112 and 123
274	O^+^ + H_2_O → H_2_O^+^ + O	3.2 × 10^–15^	132
275	O^+^ + O_2_ → O_2_^+^ + O	2.0 × 10^–17^*T*_0_^–0.4^	133
276	O_2_^+^·H_2_O (+ He) → O_2_^+^ + H_2_O (+ He)	Effective	87 ^*c*^ ^,^ ^*d*^
277	O_2_^+^·H_2_O + H_2_O → H_2_O^+^·H_2_O + O_2_	1.0 × 10^–15^	118 ^*b*^
278	O_4_^+^ + He → O_2_^+^ + O_2_ + He	3.4 × 10^–20^	74 and 134
279	O_4_^+^ + H_2_O → O_2_^+^·H_2_O + O_2_	1.7 × 10^–15^	135
280	O_4_^+^ + O → O_2_^+^ + O_3_	3.0 × 10^–16^	118
281	O_4_^+^ + O_2_ → O_2_^+^ + 2O_2_	1.0 × 10^–11^*T*_0_^–4.2^e^–5400/*T*_g_^	74 and 134
282	O_4_^+^ + O_2_(a ^1^Δ) → O_2_^+^ + 2O_2_	1.0 × 10^–16^	118 ^*b*^
283	O_4_^+^ + O_2_(b ^1^Σ) → O_2_^+^ + 2O_2_	1.0 × 10^–16^	As rxn 282

Two-body collisions – negative ions			
284	H^–^ + He → H + He + e	4.43 × 10^–17^e^–5829/*T*_g_^	136
285	H^–^ + H_2_O → OH^–^ + H_2_	4.8 × 10^–15^	112 and 137
286	OH^–^ + H → H_2_O + e	1.4 × 10^–15^	112 and 138
287	H_2_O_2_^–^ + H_2_O → OH^–^·(H_2_O) + OH	1.0 × 10^–17^	139 ^*f*^
288	O^–^ + H_2_O → OH^–^ + OH	1.4 × 10^–15^	105
289	O^–^ + O_2_ → O_2_^–^ + O	1.0 × 10^–18^	74 and 134
290	O^–^ + O_2_(a ^1^Δ) → O_2_^–^ + O	7.9 × 10^–16^e^–890/*T*_g_^	74 and 140
291	O^–^ + O_2_(a ^1^Δ) → O_3_ + e	6.1 × 10^–16^	74 and 140
292	O^–^ + O_2_(b ^1^Σ) → O_2_^–^ + O	7.9 × 10^–16^e^–890/*T*_g_^	As rxn 290
293	O^–^ + O_2_(b ^1^Σ) → O_3_ + e	6.1 × 10^–16^	74 and 140
294	O^–^ + O_3_ → O_2_^–^ + O_2_	1.0 × 10^–17^	74 and 134
295	O_2_^–^ + O → O^–^ + O_2_	8.5 × 10^–17^*T*_0_^–1.7^	141 ^*g*^
296	O_2_^–^ + O → O_3_ + e	8.5 × 10^–17^*T*_0_^–1.7^	141 ^*g*^

Three-body collisions – positive ions			
297	He^+^ + 2He → He_2_^+^ + He	1.4 × 10^–43^*T*_0_^–0.6^	142
298	H^+^·(H_2_O) + H_2_O (+ He) → H^+^·(H_2_O)_2_ (+ He)	Effective	87 and 143 ^*c*^ ^,^ ^*d*^
299	H^+^·(H_2_O)_2_ + H_2_O (+ He) → H^+^·(H_2_O)_3_ (+ He)	Effective	87 and 143 ^*c*^ ^,^ ^*d*^
300	H^+^·(H_2_O)_3_ + H_2_O (+ He) → H^+^·(H_2_O)_4_ (+ He)	Effective	87 and 143 ^*c*^ ^,^ ^*d*^
301	H^+^·(H_2_O)_4_ + H_2_O (+ He) → H^+^·(H_2_O)_5_ (+ He)	Effective	87 and 143 ^*c*^ ^,^ ^*d*^
302	H^+^·(H_2_O)_5_ + H_2_O (+ He) → H^+^·(H_2_O)_6_ (+ He)	Effective	87 and 143 ^*c*^ ^,^ ^*d*^
303	H^+^·(H_2_O)_6_ + H_2_O (+ He) → H^+^·(H_2_O)_7_ (+ He)	Effective	87 and 143 ^*b*^ ^,^ ^*c*^ ^,^ ^*d*^
304	H^+^·(H_2_O)_7_ + H_2_O (+ He) → H^+^·(H_2_O)_8_ (+ He)	Effective	est.^*e*^
305	H^+^·(H_2_O)_8_ + H_2_O (+ He) →H^+^·(H_2_O)_9_ (+ He)	Effective	est.^*e*^
306	O_2_^+^ + H_2_O (+ He) → O_2_^+^·H_2_O (+ He)	Effective	87 ^*c*^ ^,^ ^*d*^
307	O_2_^+^ + O_2_ (+ He) → O_4_^+^ (+ He)	Effective	87 ^*c*^ ^,^ ^*d*^

Three-body collisions – negative ions			
308	OH^–^ + H_2_O + He → OH^–^·(H_2_O) + He	8.0 × 10^–42^	144 ^*h*^
309	OH^–^·(H_2_O) + H_2_O + He → OH^–^·(H_2_O)_2_ + He	2.5 × 10^–43^	144 ^*h*^
310	OH^–^·(H_2_O)_2_ + H_2_O + He → OH^–^·(H_2_O)_3_ + He	1.5 × 10^–43^	144 ^*h*^
311	O^–^ + H_2_O + He → H_2_O_2_^–^ + He	1.3 × 10^–40^	139

^*a*^In s^–1^, m^3^ s^–1^ and m^6^ s^–1^ for one-, two- and three-body reactions, respectively.

^*b*^Value is estimated in reference.

^*c*^Effective rate coefficients calculated from pressure dependent rates as described by Sieck^87^ for 1 atm and a temperature range 280–350 K.

^*d*^Background gas is (humid) air in given reference. Gas efficiency factors for He background gas are not known for these reactions, but could potentially change calculated reaction rate coefficients if taken into account.

^*e*^Rate coefficients are estimated by extrapolating the coefficients *k*3000 and *A* given by Sieck *et al.*^87^ using an exponential fit, and using constant values *n* = 16, *B* = 5000, and *k*_L_ = 10^–24^.

^*f*^Value is listed as a lower limit in reference.

^*g*^Estimated branching ratio.

^*h*^Third body is H_2_O in reference.

**Table 8:** Neutral chemistry

No.	Reaction (rxn)	Rate^*a*^	Ref.
Two-body collisions			
312	He + O(^1^D) → O + He	7.0 × 10^–22^	145 ^*b*^
313	He + O(^1^S) → O + He	7.0 × 10^–22^	As rxn 312
314	He + O_2_(a ^1^Δ) → O_2_ + He	8.0 × 10^–27^	146 ^*b*^
315	He + O_2_(b ^1^Σ) → O_2_(a ^1^Δ) + He	1.0 × 10^–23^*T*_0_^0.5^	125 ^*c*^
316	2He* → He + He^+^ + e	4.5 × 10^–16^	46 and 97
317	2He* → He_2_^+^ + e	1.05 × 10^–15^	46 and 97
318	He* + He_2_* → 2He + He^+^ + e	5.0 × 10^–16^	97 ^*d*^
319	He* + He_2_* → He + He_2_^+^ + e	2.0 × 10^–15^	97 ^*d*^
320	He* + OH → OH^+^ + He + e	6.08 × 10^–16^	As rxn 321
321	He* + H_2_O → He + H_2_O^+^ + e	6.08 × 10^–16^	147 ^*e*^
322	He* + H_2_O → He + OH^+^ + H + e	1.39 × 10^–16^	147 ^*e*^
323	He* + H_2_O_2_ → He + OH^+^ + OH + e	6.08 × 10^–16^	As rxn 321
324	He* + O → O^+^ + He + e	2.54 × 10^–16^	As rxn 327
325	He* + O(^1^D) → O^+^ + He + e	2.54 × 10^–16^	As rxn 327
326	He* + O(^1^S) → O^+^ + He + e	2.54 × 10^–16^	As rxn 327
327	He* + O_2_ → O_2_^+^ + He + e	2.54 × 10^–16^	148
328	He* + O_2_(a ^1^Δ) → O_2_^+^ + He + e	2.54 × 10^–16^	As rxn 327
329	He* + O_2_(b ^1^Σ) → O_2_^+^ + He + e	2.54 × 10^–16^	As rxn 327
330	He* + O_3_ → O_2_^+^ + O + He + e	2.6 × 10^–16^	74 ^*c*^
331	He_2_* + H_2_O → 2He + H_2_O^+^ + e	2.2 × 10^–15^	149
332	He_2_* + O → O^+^ + 2He + e	3.6 × 10^–16^	As rxn 335
333	He_2_* + O(^1^D) → O^+^ + 2He + e	3.6 × 10^–16^	As rxn 335
334	He_2_* + O(^1^S) → O^+^ + 2He + e	3.6 × 10^–16^	As rxn 335
335	He_2_* + O_2_ → O_2_^+^ + 2He + e	3.6 × 10^–16^	149
336	He_2_* + O_2_(a ^1^Δ) → O_2_^+^ + 2He + e	3.6 × 10^–16^	As rxn 335
337	He_2_* + O_2_(b ^1^Σ) → O_2_^+^ + 2He + e	3.6 × 10^–16^	As rxn 335
338	He_2_* + O_3_ → O_2_^+^ + O + 2He + e	3.6 × 10^–16^	74 ^*c*^
339	H + HO_2_ → H_2_ + O_2_	5.6 × 10^–18^	88
340	H + HO_2_ → 2OH	7.2 × 10^–17^	88
341	H + HO_2_ → H_2_O + O	2.4 × 10^–18^	88
342	H + H_2_O_2_ → H_2_O + OH	1.7 × 10^–17^e^–1800/*T*_g_^	150
343	H + H_2_O_2_ → H_2_ + HO_2_	2.8 × 10^–18^e^–1890/*T*_g_^	150
344	H + O_3_ → OH + O_2_	1.4 × 10^–16^e^–470/*T*_g_^	151 and 152
345	H_2_ + OH → H_2_O + H	4.27 × 10^–19^*T*_0_^2.41^e^–1240/*T*_g_^	153
346	H_2_ + O(^1^D) → OH + H	1.2 × 10^–16^	88
347	H_2_ + O(^1^S) → OH + H	1.2 × 10^–16^	As rxn 346
348	2OH → H_2_O + O	6.2 × 10^–20^*T*_0_^2.6^e^945/*T*_g_^	88
349	OH + HO_2_ → O_2_ + H_2_O	4.8 × 10^–17^e^250/*T*_g_^	88, 154 and 155
350	OH + H_2_O_2_ → HO_2_ + H_2_O	2.9 × 10^–18^e^–160/*T*_g_^	88
351	OH + O → H + O_2_	2.4 × 10^–17^e^110/*T*_g_^	88, 156 and 157
352	OH + O(^1^D) → O_2_ + H	2.4 × 10^–17^e^110/*T*_g_^	As rxn 351
353	OH + O(^1^S) → O_2_ + H	2.4 × 10^–17^e^110/*T*_g_^	As rxn 351
354	OH + O_3_ → O_2_ + HO_2_	1.7 × 10^–18^e^–940/*T*_g_^	88
355	H_2_O + O(^1^D) → 2OH	1.63 × 10^–16^e^60/*T*_g_^	151
356	H_2_O + O(^1^S) → O + H_2_O	4.5 × 10^–17^	158
357	H_2_O + O(^1^S) → O(^1^D) + H_2_O	1.5 × 10^–16^	158
358	H_2_O + O(^1^S) → 2OH	3.05 × 10^–16^	158
359	H_2_O + O_2_(a ^1^Δ) → O_2_ + H_2_O	4.8 × 10^–24^	151
360	H_2_O + O_2_(b ^1^Σ) → O_2_ + H_2_O	3.9 × 10^–18^e^125/*T*_g_^	151
361	HO_2_ + O → OH + O_2_	2.7 × 10^–17^e^224/*T*_g_^	88 and 155
362	HO_2_ + O(^1^D) → OH + O_2_	5.2 × 10^–16^	As rxn 364
363	HO_2_ + O(^1^S) → OH + O_2_	5.2 × 10^–16^	As rxn 364
364	H_2_O_2_ + O(^1^D) → H_2_O + O_2_	5.2 × 10^–16^	159
365	H_2_O_2_ + O(^1^S) → H_2_O + O_2_	5.2 × 10^–16^	As rxn 364
366	O + O(^1^D) → 2O	8.0 × 10^–18^	160
367	O + O(^1^S) → 2O	3.33 × 10^–17^e^–300/*T*_g_^	125 and 161 ^*d*^
368	O + O(^1^S) → O(^1^D) + O	1.67 × 10^–17^e^–300/*T*_g_^	125 and 161 ^*d*^
369	O(^1^D) + O_2_ → O + O_2_	6.4 × 10^–18^	88
370	O(^1^D) + O_2_ → O + O_2_(b ^1^Σ)	2.56 × 10^–17^	88
371	O(^1^D) + O_3_ → 2O_2_	1.2 × 10^–16^	88
372	O(^1^D) + O_3_ → O_2_ + 2O	1.2 × 10^–16^	88
373	O(^1^S) + O_3_ → 2O_2_	1.2 × 10^–16^	As rxn 371
374	O(^1^S) + O_3_ → O_2_ + 2O	1.2 × 10^–16^	As rxn 372
375	O_2_ + O_2_(a ^1^Δ) → 2O_2_	3.0 × 10^–24^e^–200/*T*_g_^	88
376	O_2_ + O_2_(b ^1^Σ) → O_2_ + O_2_(a ^1^Δ)	3.6 × 10^–23^*T*_0_^0.5^	125
377	2O_2_(a ^1^Δ) → O_2_(b ^1^Σ) + O_2_	1.8 × 10^–24^*T*_0_^3.8^e^700/*T*_g_^	162 and 163
378	O_2_(a ^1^Δ) + O_3_ → O + 2O_2_	5.2 × 10^–17^e^–2840/*T*_g_^	151
379	O_2_(b ^1^Σ) + O_3_ → O + 2O_2_	3.5 × 10^–17^e^–135/*T*_g_^	151

Three-body collisions			
380	2He + He* → He + He_2_*	2.0 × 10^–46^	164
381	He + He* + H_2_O → H_2_O^+^ + 2He + e	1.48 × 10^–41^	147 ^*e*^
382	He + He* + O → O^+^ + 2He + e	8.2 × 10^–42^	As rxn 385
383	He + He* + O_2_ → O_2_^+^ + 2He + e	8.2 × 10^–42^	165
384	He + H + H → H_2_ + He	6.04 × 10^–45^*T*_0_^–1.0^	150 and 152 ^*f*^
385	He + H + OH → H_2_O + He	9.23 × 10^–44^*T*_0_^–1.527^e^–185/*T*_g_^	166 ^*g*^
386	He + H + O → OH + He	4.36 × 10^–44^*T*_0_^–1.0^	167 ^*c*^
387	(He+) H + O_2_ → HO_2_ (+He)	Effective	88 ^*h*^ ^,^ ^*i*^
388	(He+) 2OH → H_2_O_2_ (+He)	Effective	88 ^*h*^ ^,^ ^*j*^
389	He + 2O → O_2_ + He	3.99 × 10^–47^e^900/*T*_g_^	167 ^*k*^
390	He + O + O_2_ → O_3_ + He	3.66 × 10^–46^*T*_0_^–2.6^	88 ^*l*^

^*a*^In m^3^ s^–1^ and m^6^ s^–1^ for two- and three-body collisions, respectively.

^*b*^Value in an upper limit in reference.

^*c*^Estimated value in reference.

^*d*^Estimated branching ratio.

^*e*^Branching ratios taken from Sanders.^168^

^*f*^Third body is Ar instead of He in reference. The gas efficiency factor is assumed to be 1.

^*g*^Third body is Ar instead of He in reference. The gas efficiency factor is assumed to be 0.65. This factor is calculated by dividing reaction rate coefficients for He and Ar as background gases for the same reaction measured by Zellner *et al.*^169^

^*h*^Effective rate coefficients calculated from pressure dependent rates for 1 atm and fitted by an Arrhenius expression in the temperature range 280–350 K.

^*i*^Third body is N_2_ instead of He in reference. The gas efficiency factor is assumed to be 0.43. This factor is calculated by dividing reaction rate coefficients for He and N_2_ as background gases for the same reaction measured by Hsu *et al.*^170^

^*j*^Recommended rate coefficient in reference is for N_2_ background gas instead of He. We apply a gas efficiency factor of 0.41 to the low-pressure limit reaction rate coefficient to account for this. This factor is calculated by dividing the room temperature rate coefficient from the given reference for He background gas (measured by Forster *et al.*^171^) by the recommended value (measured by Fulle *et al.*^172^).

^*k*^Third body is Ar instead of He in reference. The gas efficiency factor is assumed to be 0.77. This factor is calculated by dividing reaction rate coefficients for He and Ar as background gases for the same reaction measured by Campbell and Thrush.^169^

^*l*^Third body is N_2_ instead of He in reference. The gas efficiency factor is assumed to be 0.61. This factor is calculated by dividing reaction rate coefficients for He and N_2_ as background gases for the same reaction measured by Lin and Leu.^173^

Table 8 shows reaction rate coefficients for collisions between ions and neutrals. Similar to the description of ion-neutral reactions in Table 7, a number reactions rates in Table 8, including neutral reactions of several O and H containing species, are specified as “effective”. Data to calculate rate coefficients for these reactions has generally been taken from the IUPAC chemical kinetics database.^88^

For the calculation of “effective” decay rates, and generally for three-body processes, we have multiplied the three-body rate coefficient from the respective sources by a background gas dependent efficiency factor, if available, where the collider gas is different from He in the reference. This accounts for the fact that He is a less effective quencher compared to other background gases. More details for specific reactions are denoted in footnotes.

In addition to the He–H_2_O chemistry, we also include oxygen neutrals and ions. This makes it possible to investigate the influence of O_2_ impurities at low H_2_O contents on the plasma chemistry. In contrast to Liu *et al.*, we do not include the ions HeH^+^, H^+^, and H_2_^+^ due to their generally low densities compared to the more abundant protonated water clusters under all investigated conditions, and the fact that in this work, the focus lies on the investigation of the neutral particle dynamics.

##### B. List of equipment

The equipment used for the investigations in this work is listed in Table 9.

**Table 9:** Equipment used in different experimental setups for power coupling into the plasma and acquiring absorption spectra

Equipment	O FTAS	OH UV-BBAS I	OH UV-BBAS II
Power supply	Tabor WS8352	Advanced Energy RFX-600	TTi TGA 121104
	IFI SCCX100		Coaxial Power Systems RFG150
Matching box	Coaxial Power Systems MMN150	Coaxial Power Systems MMN150	Coaxial Power Systems MMN150
Voltage probe	PMK-14KVAC	Tektronix P5100A	PMK-14KVAC
Oscilloscope	Lecroy Wavejet 354A	Teledyne LeCroy HDO4104	Agilent DSO-X-2004-A
Light source	DESIRS beamline^57^ (Soleil)	Energetiq LDLS	UVTOP-305-FW-TO18 (Roithner Lasertechnik GmbH)
Spectrometer	DESIRS beamline^57^ (Soleil)	Princeton Instruments IsoPlane SCT320	Andor SR-500i
Detector	DESIRS beamline^57^ (Soleil)	Hamamatsu S-3904	Andor Newton 940
